# Maturation strategy influences expression levels and cofactor occupancy in Fe–S proteins

**DOI:** 10.1007/s00775-022-01972-1

**Published:** 2022-12-17

**Authors:** Melissa Jansing, Steffen Mielenbrink, Hannah Rosenbach, Sabine Metzger, Ingrid Span

**Affiliations:** 1 Institut für Physikalische Biologie, Heinrich-Heine-Universität Düsseldorf, Universitätsstr. 1, 40225 Düsseldorf, Germany; 2 MS-Platform Biocenter, Cluster of Excellence on Plant Science (CEPLAS), University of Cologne, Zülpicher Strasse 47B, 50674 Cologne, Germany; 3 Bioanorganische Chemie, Department Chemie und Pharmazie, Friedrich-Alexander-Universität Erlangen-Nürnberg, Egerlandstr. 1, 91058 Erlangen, Germany

**Keywords:** Fe–S protein biogenesis, Fe–S protein overproduction, Fe–S protein maturation, Iron–sulfur cluster pathway, Chemical reconstitution

## Abstract

Iron–sulfur clusters are ubiquitous cofactors required for fundamental biological processes. Structural and spectroscopic analysis of Fe–S proteins is often limited by low cluster occupancy in recombinantly produced proteins. In this work, we report a systematic comparison of different maturation strategies for three well-established [4Fe–4S] proteins. Aconitase B, HMBPP reductase (IspH), and quinolinate synthase (NadA) were used as model proteins as they have previously been characterized. The protein production strategies include expression of the gene of interest in BL21(DE3) cells, maturation of the apo protein using chemical or semi-enzymatic reconstitution, co-expression with two different plasmids containing the iron–sulfur cluster (*isc*) or sulfur formation (*suf*) operon, a cell strain lacking IscR, the transcriptional regulator of the ISC machinery, and an engineered “SufFeScient” derivative of BL21(DE3). Our results show that co-expression of a Fe–S biogenesis pathway influences the protein yield and the cluster content of the proteins. The presence of the Fe–S cluster is contributing to correct folding and structural stability of the proteins. In vivo maturation reduces the formation of Fe–S aggregates, which occur frequently when performing chemical reconstitution. Furthermore, we show that the in vivo strategies can be extended to the radical SAM protein ThnB, which was previously only maturated by chemical reconstitution. Our results shed light on the differences of in vitro and in vivo Fe–S cluster maturation and points out the pitfalls of chemical reconstitution.

**Graphical abstract:**

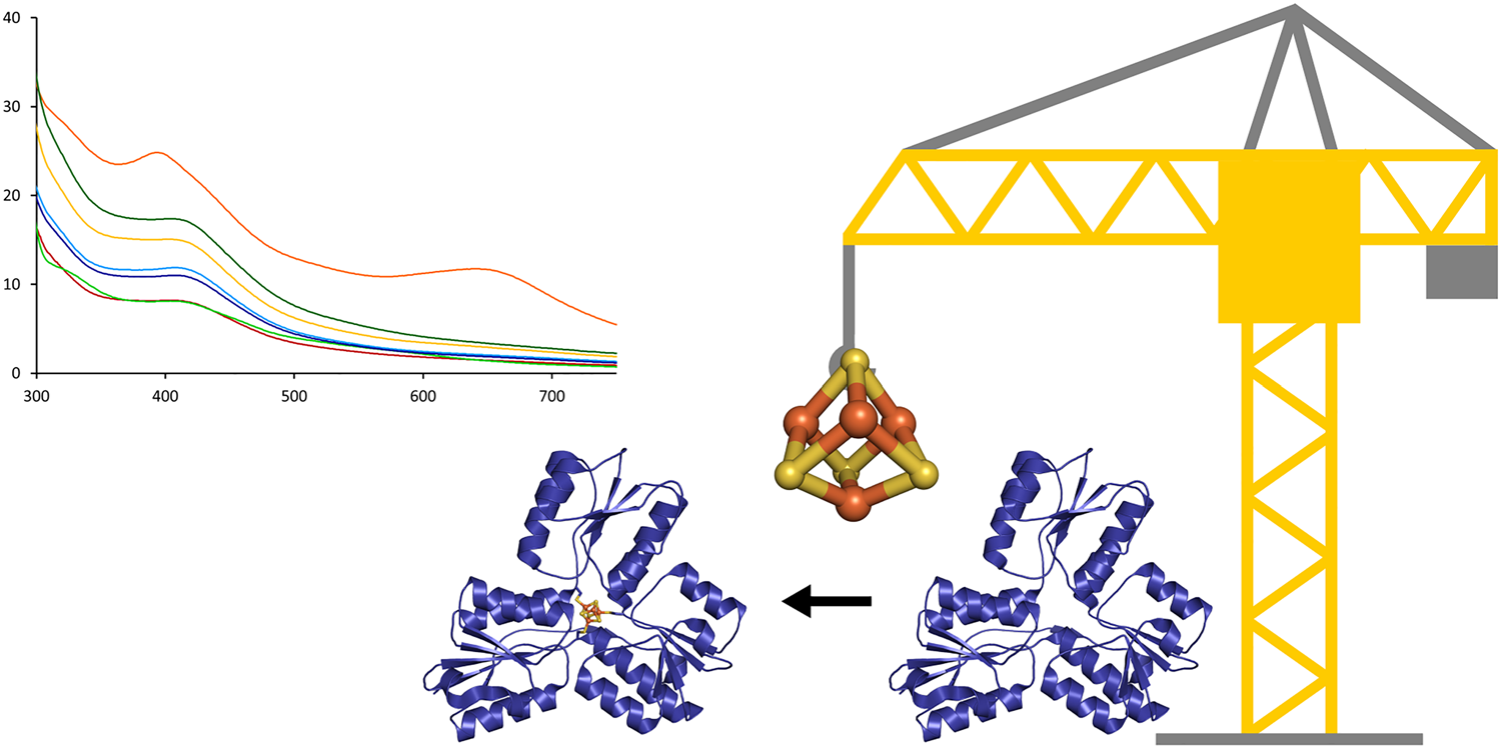

**Supplementary Information:**

The online version contains supplementary material available at 10.1007/s00775-022-01972-1.

## Introduction

Fe–S clusters are ancient and ubiquitous cofactors in proteins. They are involved in many essential biological processes, including oxidative respiration, photosynthesis, hydrogen production, nitrogen fixation, and DNA replication/repair. The most common species found in proteins are the rhombic [2Fe–2S] and the cubic [4Fe–4S] forms. They are predominantly coordinated by cysteine residues, however, other amino acids can also serve as ligands [1]. The most common role of Fe–S proteins in nature is as electron transfer proteins. Furthermore, the Fe–S cluster also plays important roles in providing stability to protein structures, regulating gene expression, non-redox catalysis, repair and processing of nucleic acids, regulation of cellular processes, and iron homeostasis [2–4].

Fe–S cofactors have played a significant role in the metabolism of the primordial cells, which were formed in an atmosphere with an extremely low oxygen concentration [5]. Under these conditions, Fe(II) was stable in aqueous solutions and the versatile and stable Fe–S clusters evolved to the most common redox cofactor. With increasing concentration of molecular oxygen in the atmosphere, Fe(II) was oxidized by O_2_ to Fe(III), which is insoluble in aqueous solutions. With the rise of atmospheric oxygen, some organisms have evolved to utilize copper proteins instead of Fe–S proteins for the same functions [6]. Despite the need to protect the cofactors from oxygen, Fe–S proteins remain ubiquitous cofactors in nature and their key role in many biological processes makes them the focus of a large research community [3].

A major challenge in the field is to obtain large amounts of homogenous protein sample of high purity and at high concentrations. The production of recombinant proteins is a straightforward approach to obtain large quantities of the desired protein. *Escherichia coli* is one of the organisms of choice for the production of recombinant proteins [7]. For the spectroscopic, structural, and functional characterization of Fe–S proteins, it is also crucial to produce protein samples with a fully occupied cofactor [8]. The sensitivity of Fe–S proteins towards oxygen requires special attention during gene expression, protein isolation, and purification. Gene expression occurs in the cytosol with a reducing environment and virtually no free molecular oxygen, so in most cases, the cells can be cultivated under aerobic conditions without an impact on the Fe–S proteins. However, cultivation of cells under shaking with low speed as well as expression under anaerobic conditions have been shown to have a beneficial effect on Fe–S protein production [9]. *E. coli* is a facultative anaerobic bacterium [10], thus, it can also be cultivated at low oxygen concentrations. After cell lysis, the Fe–S proteins are exposed to molecular oxygen and their cofactor decomposes rapidly in most cases due to the insolubility of ferric ions. Some members of the Fe–S protein family are capable of stabilizing protein-bound ferric ions, such as rubredoxins [11, 12], or the clusters are protected from the environment by the protein, such as some ferredoxins [13], resulting in no or slow degradation of the Fe–S cluster under aerobic conditions. However, in most cases, isolation of Fe–S proteins under aerobic conditions leads to protein in the apo form or with partial cluster occupancy.

Another important consideration is the assembly and transfer of the cofactors to recombinantly produced proteins using the Fe–S biogenesis pathways of the host. Several different machineries for the maturation of Fe–S proteins are known. The iron–sulfur cluster (ISC) machinery is used for maturating housekeeping proteins under standard growth conditions in bacteria and eukaryotic mitochondria (reviewed in Lill 2009 [14]) and the sulfur formation (SUF) machinery is used under stress conditions in bacteria and chloroplasts of eukaryotes (reviewed in Bai et al. 2018 [15]). The SUF pathway is the sole machinery for Fe–S cluster biogenesis in archaea, cyanobacteria, and many Gram-positive, thermophilic and pathogenic bacteria [16]. In addition, the nitrogen fixation (NIF) machinery is responsible for Fe–S cluster assembly in nitrogen fixing organisms such as *Azotobacter vinelandii* [17]. Furthermore, the cysteine sulfinate desulfinase (CSD) from *E. coli* was reported to have components similar to the ISC or SUF system, but lacks a scaffold protein [18]. Maturation of cytosolic and nuclear Fe–S proteins in eukaryotes is performed by the cytosolic iron–sulfur cluster assembly (CIA) system (reviewed in Sharma et al. 2010 [19]).

**Fig. 1:**
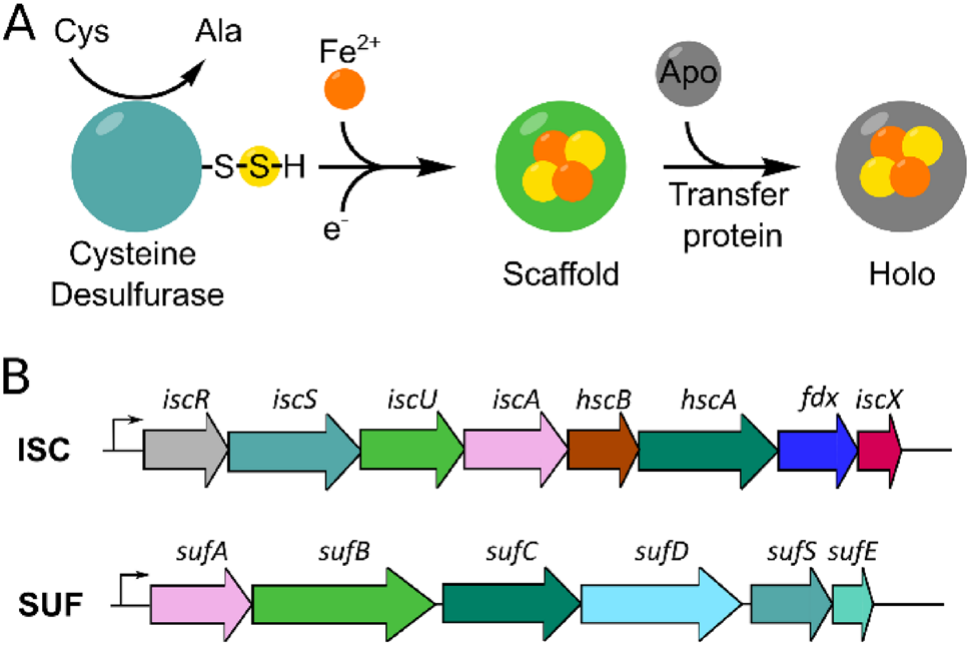
Iron–sulfur cluster biosynthesis in *Escherichia coli*. **A** Simplified representation of Fe–S cluster biogenesis [14]. **B** Overview of the ISC and SUF machinery producing gene clusters from *E. coli*. Each operon contains proteins with similar functions, which are shown in the same color

Several systems for the biogenesis of Fe–S clusters have been identified in bacteria and eukaryotes (Fig. 1A). Despite the structural diversity of the protein constituents of these four machineries, these different machineries follow common biosynthetic routes (reviewed in Lill 2009 and Braymer et al. 2021 [14, 20]). The first step of Fe–S cluster biogenesis requires a pyridoxal 5′-phosphate-dependent cysteine desulfurase, which converts the substrate L-cysteine into L-alanine, resulting in the formation of a persulfide on a conserved cysteine residue of the enzyme. This persulfide may be transferred to conserved cysteine residues of the scaffold proteins directly, or through an intermediary helper protein. Apart from sulfur, de novo Fe–S cluster synthesis requires iron, yet a dedicated iron donor protein or an iron chaperone has not been unambiguously identified in any of the biogenesis systems. Further, an electron donor is required for the assembly of the Fe–S clusters. Scaffold proteins serve as a platform for the de novo assembly of Fe–S clusters. These proteins combine the ability to assemble clusters efficiently with the capacity to pass the cofactors on to either apo target proteins or trafficking factors. The central importance of the Fe–S machineries is highlighted by the fact that so far, no biological example has been described for de novo synthesis of Fe–S clusters occurring directly on target apoproteins.

For the recombinant production of Fe–S proteins, it must be considered that the recombinant gene is expressed to an abnormally and excessively high level, whereas the level of the Fe–S assembly proteins remains unchanged. Thus, the machinery may not be capable of keeping up with the production of the cofactor, which results in incomplete maturation. The amount of protein-bound Fe–S cluster in partially occupied proteins can be enhanced by chemical reconstitution, which is performed by treating the protein with an inorganic iron and sulfide source in the presence of a reductant [21]. This approach contains certain pitfalls, in cases where the nature of the native Fe–S cluster has not been established, chemical reconstitution can result in incomplete cluster formation or the assembly of artifacts [22, 23]. A more elegant variation of the reconstitution reaction is a semi-enzymatic approach that involves the protein cysteine desulfurase and L-cysteine instead an inorganic sulfur source [24].

Previous studies have shown that co-expression of genes encoding Fe–S proteins with the *isc* or *suf* operon (Fig. 1B) have resulted in higher protein yields and increased cofactor occupancy [25, 26]. Furthermore, it was reported that the deletion of the gene encoding the transcriptional regulator of the ISC machinery, *iscR*, in *E. coli* BL21(DE3) cells results in an enhanced expression of the *isc* operon providing an alternative to plasmid-based overexpression [27]. Interestingly, it was shown that *E. coli* BL21(DE3), a strain routinely used to overproduce [Fe–S] cluster-containing proteins, has a nonfunctional SUF pathway [7]. However, an engineered strain was successfully generated, in which an intact SUF pathway was restored. This engineered derivative of BL21(DE3), also referred to as “SufFeScient” or Suf^++^, was suitable for enhanced large-scale synthesis of an [Fe–S] cluster-containing protein. Each approach has been successfully used for one or several proteins [21, 24, 26, 28, 29], however, systematic studies comparing all these strategies for the same protein have not yet been reported. A straightforward approach would be to use the biogenesis pathway that is biologically relevant, which is only possible for prokaryotic proteins. In addition, the co-expression of two plasmids may result in an additional stress for the host cells and, therefore, reduce the productivity of protein synthesis.

In this study, we investigate seven different strategies for the production and maturation of recombinant Fe–S proteins. *E. coli* is one of the organisms of choice for the production of recombinant proteins and it has become the most popular expression platform [30]. In addition, *E. coli* variants with increased expression of the genes encoding for the Fe–S cluster biosynthesis machineries have been engineered, providing the ideal platform to investigate Fe–S maturation of bacterial proteins. Our results show that selection of the appropriate strategy increases protein yield and Fe–S cluster content of proteins. We use electronic absorption spectroscopy (EAS) and electronic circular dichroism (ECD) spectroscopy to assess the amount of Fe–S cluster bound to the protein of interest, to compare the spectral properties of the different approaches, to reveal excess iron, and to detect impurities with absorption features. In addition, we quantified the Fe–S cluster content of the samples using inductively coupled plasma mass spectrometry (ICP-MS). As target proteins, we have selected three well-characterized proteins that contain [4Fe–4S] clusters coordinated by three cysteine residues of the protein backbone, which have been investigated by spectroscopy and crystallography. Thus, the spectral properties corresponding to the [4Fe–4S] cluster in the protein of interest have been established. The function and biological relevance of the three target proteins will be briefly described in the following paragraph.

The enzyme 4-hydroxy-3-methylbut-2-enyl diphosphate reductase (IspH) from *E. coli* produces the universal precursors of terpenes in the methylerythritol phosphate isoprenoid biosynthesis using its 2H^+^/2e^–^ reductase activity [29]. The structure of the cluster in IspH has been discussed controversially for some time, however, crystal structures of the enzyme in complex to the substrate have unambiguously identified the [4Fe–4S] cluster as the cofactor of the active enzyme [31]. Quinolinate synthase A (NadA) from *E. coli* plays an important role in the synthesis of nicotinamide adenine dinucleotide (NAD), the primary biological cofactor for oxidation–reduction reactions [32]. The biosynthesis of NAD varies considerably between prokaryotes and eukaryotes. *E. coli* and most other prokaryotes generate quinolinic acid via a unique condensation reaction between dihydroxyacetone phosphate (DHAP) and iminosuccinate (IS) in an “anaerobic” pathway. This reaction requires the concerted efforts of two enzymes, quinolinate synthetase (NadA), and aspartate oxidase (NadB). Characterization of the protein by electronic absorption, electron paramagnetic resonance (EPR), and Mössbauer spectroscopies shows unequivocally that NadA contains one [4Fe–4S] cluster per polypeptide [33]. The major bifunctional aconitase in *E. coli* (AcnB) serves as either an enzymatic catalyst or a mRNA-binding posttranscriptional regulator, depending on the status of its iron–sulfur cluster. In the presence of iron, AcnB contains an [4Fe–4S] cluster and functions as an enzyme in the citric acid and glyoxylate cycles. Under oxidative stress conditions and iron starvation, the [4Fe–4S] cluster is interconverted to an [3Fe–4S] cluster and the protein functions as a nucleic acid binding protein that binds to its own mRNA inducing a positive regulation [34–36]. The crystal structure of an inactive porcine heart mitochondrial aconitase hosting a [3Fe–4S] cluster reveals that the cluster is ligated by three cysteine residues [37]. The fourth coordination partner in the active form of the aconitase with a [4Fe–4S] cluster is proposed to be the citrate substrate [35].

In addition to the three well-characterized enzymes, we selected another system that, to our knowledge, has not been maturated in vivo so far. *Bacillus thuringiensis* Thurincin B (ThnB) is a member of the radical SAM enzyme family that are reported to be involved in the maturation process of sactipeptides [38]. Sactipeptides represent a new class of natural products featuring promising antimicrobial activities [39]. However, the role of these peptides within the host organism remains unknown [40]. The first step in the biosynthesis of sactipeptides is the formation of the characteristic cysteine-sulfur to α-carbon thioether linkage in the precursor peptide [40, 41]. It has been shown that this reaction is catalyzed by enzymes that incorporate two Fe–S clusters and a SAM cofactor. These enzymes share a characteristic CX_3_CX_2_C motif, which is capable of binding one catalytic [4Fe–4S] cluster that is required for the reductive cleavage of SAM into methionine and a 5′-deoxyadenosyl (5′-dA) radical [42]. The function of a second [4Fe–4S] cluster, hosted by radical SAM enzymes and studied, e.g., in the radical SAM enzyme family members subtilisin A (AlbA) and sporulation killing factor (SkfB) is presumably electron transfer during the process of thioether bond formation [40, 43–46].

The production of correctly assembled Fe–S cluster proteins remains a major challenge in the bioinorganic chemistry field. In this study, we compare seven different strategies for protein production and maturation: (i) expression in BL21(DE3); (ii) expression in BL21(DE3) and chemical reconstitution; (iii) expression in BL21(DE3) and semi-enzymatic reconstitution; (iv) co-expression in BL21(DE3) with pISC; (v) co-expression in BL21(DE3) with pSUF; (vi) expression in BL21(DE3) ∆*iscR*; and expression in BL21(DE3) Suf^++^ cells. Hereby, gene expression was always performed under aerobic conditions, cell lysis and all further steps under anaerobic conditions. The target proteins IspH, NadA, and AcnB contain one [4Fe–4S] cluster ligated by three cysteine residues and originated from *E. coli*, which hosts two Fe–S biogenesis pathways: the ISC and the SUF machinery [47]. We also studied cluster incorporation into the radical SAM enzyme *B. thuringiensis* ThnB that contains two Fe–S clusters, one of them with an open coordination site for the SAM cofactor. Gram-positive bacteria, including *B. subtilis*, have been reported to rely solely on the SUF pathway [48]. Therefore, we paid particular attention on how the co-expression with the ISC or SUF machinery from *E. coli* influences the expression of the radical SAM protein from *B. thuringiensis*.

Most structural and functional investigations rely on heterologous enzyme production. Several barriers for producing active enzymes in non-native hosts have been proposed, including inefficient transcription or translation. These obstacles can be overcome by engineering suitable constructs to increase the efficiency of gene expression. However, Fe–S enzymes face additional barriers, such as the incompatibility with foreign Fe–S biogenesis pathways. A recent study reported a systematic investigation of the ability of two Fe–S enzymes to retain activity within a foreign host [49]. This study uses cellular assays to identify barriers impeding Fe–S activity in non-native prokaryotic hosts and highlights the importance for further systematic studies of the maturation of Fe–S enzymes.

## Materials and methods

### Construct design and cloning

The genes *ispH*, *nadA*, *acnB* and *thnB* were obtained as DNA strings from GeneArt (Thermo Fisher Scientific, Waltham, MA, USA). The plasmid pET16bTEV was linearized using the primers 5′-TAA CTA GCA TAA CCC CTT GGG GC-3′ and 5′-CATA TGT CCC TGA AAA TAC AGG TTT TCA TGG C-3′. All polymerase chain reactions were performed with the Phusion High-Fidelity DNA Polymerase (New England Biolabs, Ipswich, MA, USA). The plasmids pET16bTEV-*ispH*, pET16bTEV-*nadA*, pET16bTEV-*acnB* and pET16TEV-*thnB* were engineered using the In-Fusion HD cloning kit (Takara Bio, Kisatsu, Japan) and verified by sequencing (Microsynth Seqlab, Göttingen, Germany).

### Gene expression and protein isolation

For gene expression in *E. coli* BL21(DE3) overnight starting cultures containing one of the plasmids hosting the genes *ispH*, *nadA*, *acnB*, or *thnB* were used to inoculate 2 × yeast extract (2YT) media at 1 % (v/v). For a list of all plasmids used in this study, see Table S1. 2YT media was supplemented with a final concentration of 100 µg/ml ampicillin and the cells were cultivated under shaking at 37 ℃ and 160 rpm. After inoculation, the growth of the cell culture was monitored by measuring the optical density at 600 nm (OD_600_). A growth curve showed that the cell cultures reach the mid-log phase at an OD_600_ of 2 and the stationary phase at an OD_600_ of 4 in 2YT media. The expression of the gene of interest was induced by adding 0.5 mM isopropyl-*β*-D-thiogalactopyranoside (IPTG) at a mid-log phase because the culture is growing fast and protein translation is maximal.

For the maturation of the proteins using the operons of the Fe–S cluster assembly machineries, gene expression was performed in the cell strain *E. coli* BL21(DE3) ∆i*scR* [28], *E. coli* BL21(DE3) Suf^++^ [7], and *E. coli* BL21(DE3) containing either pACYC184*iscS-fdx* [29] (pISC) or pACYC-Duet-1-*suf* (pSUF) [26]. For a list of all cell strains used in this study, see Table S2. Overnight starting cultures of the cell strain containing the plasmid encoding the target protein were used to inoculate terrific broth (TB) media at 1% (v/v). TB media was supplemented with kanamycin (50 µg/ml), ampicillin (100 µg/ml), or chloramphenicol (25 µg/ml) as needed, as well as ferric ammonium citrate (2 mM final concentration). Cells were cultivated aerobically at 37 °C and 160 rpm until the OD_600_ reached 2. Subsequently, the gene expression was induced using 0.5 mM IPTG. To facilitate Fe–S cluster assembly 2 mM L-cysteine were added. For the expression of the constructs in *E. coli* BL21(DE3) Suf^++^ ferric ammonium citrate and L-cysteine were added in a final concentration of 1 mM. All cultures were incubated at 25 °C and 140 rpm for 20 h following induction. Cells were harvested by centrifugation for 10 min at 6000 × g and 4 °C. For cell lysis cells were resuspended in 50 mM Tris pH 8.0, 150 mM NaCl containing EDTA-free cOmplete™ Protease Inhibitor Cocktail Tablets (Roche, Basel, Switzerland). The mixture was stirred for 20 min at room temperature under anaerobic conditions, then covered with argon to maintain the anaerobic environment during cell lysis. The cell suspension was then sonicated (Bandelin electronic, Berlin, Germany) for 20 min with an amplitude of 60 % and a pulse of 1 s every 3 s using VS70/T sonotrode. Argon covered lysates were clarified by centrifugation at 40,000 × g.

### Anaerobic protein purification

Protein purification and all following procedures were carried out under strictly anaerobic condition in an anaerobic chamber with < 2 ppm O_2_. Isolation and purification of the protein was performed using immobilized metal affinity chromatography (IMAC). Therefore, protein lysates were applied to a HisTrap excel Ni–NTA column (Cytiva) that was equilibrated in wash buffer (50 mM Tris pH 8.0, 150 mM NaCl) using an ÄKTA start system (GE Healthcare, Little Chalfont, UK). The column was then washed with wash buffer containing 50 mM imidazole to remove unspecifically bound proteins and the target protein was eluted with buffer containing 250 mM imidazole. Fractions containing the target protein were pooled, dialyzed against wash buffer, and their purity was analyzed by sodium dodecyl sulfate polyacrylamide gel electrophoresis (SDS-PAGE). Protein concentrations of the protein samples were determined using the Bradford method [50].

### Chemical reconstitution

For the first step of the chemical reconstitution reaction, the reduction of the as-isolated protein with dithiothreitol (DTT), 100 µM protein solution was incubated with 1 mM DTT for 1 h on ice and then supplemented with 600 µM ferric ammonium citrate (FAC) and 600 µM Li_2_S for the proteins with one [4Fe–4S] cluster. In case of ThnB, which contains two [4Fe–4S] clusters, 1.2 mM FAC and 1.2 mM Li_2_S were added. After an incubation time of 1 h, the protein was applied to a PD-10 Desalting Column (GE Healthcare, Little Chalfont, UK) that was equilibrated with 50 mM HEPES, pH 7.5, 100 mM KCl to separate the protein from excess iron and sulfide. Finally, the volume of the protein solution was adjusted to 500 µl.

### Semi-enzymatic reconstitution

For the semi-enzymatic reconstitution reaction, 100 µM protein solution was incubated on ice with 1 mM DTT for 1 h. Then, 20 µM *E. coli* cysteine desulfurase IscS, 5 mM L-cysteine, and 600 µM FAC were added to the protein followed by incubation for 1 h. Subsequently, excess reagents were removed using a PD-10 Desalting Column (GE Healthcare, Little Chalfont, UK) equilibrated with 50 mM HEPES, pH 7.5; 100 mM KCl. Finally, the volume of the protein solution was adjusted to 500 µl.

### Western blot analysis

Western blot analysis was performed to confirm the expression of the His_10_-tagged constructs. Samples of the expression cultures were normalized to an OD_600_ of 1 and centrifuged for 10 min at 16,000 × g. The obtained cell pellet was resuspended in SDS loading dye and heated at 95 °C for 15 min. Protein samples were separated through SDS-PAGE and subsequently transferred to a PVDF membrane at 330 mA for 1 h using a wet blotting system (BioRad). Blocking of the membrane was performed for 1 h with rocking in a 5 % milk solution in TBS-T. The membrane was washed three times for 10 min in TBS-T and then incubated with a conjugated anti-His antibody in a dilution of 1:5000 in TBS-T for 1 h at room temperature or overnight at 4 °C. After washing three times with TBS-T, the membrane was developed with a chemiluminescence reagent (BioRad).

### Determination of metal content

To obtain the metal-to-monomer ratio of the isolated samples, we determined the protein concentration using the Bradford method [50] and measured the iron content using inductively coupled plasma mass spectroscopy. Samples were prepared by precipitating 10 µM of freshly, anaerobically isolated protein with 3 % trace-metal grade nitric acid. Precipitate was removed by centrifugation for 30 min at 15,000 × *g* and the sample was then transferred to a metal-free centrifugation tube (VWR, Radnor, PA, USA). Measurements of the iron content were performed using an Agilent 7700 ICP-MS (Agilent Technologies, Waldbronn, Germany) in the Biocenter MS-Platform at the University of Cologne. The measurements were done by strictly following the manufacturer’s instructions using He in the collision cell mode to minimize spectral interferences. Measurements were performed in technical triplicates and the presented data has an *r*^2^ value of 0.999.

### Electronic absorption spectroscopy

Electronic absorption spectra were collected using a Cary-60 spectrophotometer (Agilent Technologies, Ratingen, Germany) with 1 nm bandwidth, a scanning speed of 120 nm/min, and a 1 cm path length quartz cuvette at room temperature.

### Circular dichroism (CD) spectroscopy

CD spectra were collected using a Jasco J-815 Circular dichroism spectrometer (Jasco Germany, Pfungstadt, Germany) with a scanning speed of 100 nm/min and a bandwidth of 5 nm in a 2 mm path length quartz cuvette. An average of 20 scans was collected at 20 °C with 1 nm resolution. The spectra were processed with the Spectra Analysis software using the Adaptive-Smoothing function with the following parameters: convolution width 15, noise deviations 1. CD spectra of HEPES and Tris buffer were subtracted from protein spectra prior to the calculation of the molar ellipticity and plotting the data. Samples for CD spectroscopy were in a concentration range between 150 and 300 µM.

### Tobacco etch virus (TEV) cleavage

To remove the His_10_-tag from a sample of AcnB isolated from *E. coli* BL21(DE3) a TEV cleavage was performed. After addition of 0.5 mM EDTA and 1 mM DTT, the tag was cleaved by incubating the protein sample with TEV protease in a ratio of 1:100 at 4 °C while gently rocking overnight. The reaction components were removed by dialysis in buffer containing 50 mM Tris pH 8.0 and 150 mM NaCl, before applying the protein solution to a HisTrap excel column (Cytiva) to separate uncleaved protein, the His_10_-tag, and the protease from the target protein. Fractions containing the target protein were pooled.

### Aconitase activity assays

The aconitase activity was assayed with the Aconitase Assay kit (Sigma-Aldrich MAK337). Therefore, the conversion of citrate to isocitrate by the aconitase enzyme is measured. The assay reaction mix includes the substrate citrate, NADP and MTT dye, which following the generation of isocitrate is converted to an intensely violet colored form with an absorption maximum at 565 nm. As a first step to determine the activity of aconitase samples, an isocitrate standard curve was generated according to the manufacturer’s instructions. Subsequently, the activity of AcnB isolated from *E. coli* strains BL21(DE3) and BL21(DE3) ∆*iscR* as well as chemically and semi-enzymatically reconstituted AcnB was measured. The absorption was measured once after an incubation time of 10 min and a second time after an incubation time of 30 min. All samples had a protein concentration of 1 µM and all experiments were conducted in duplicates. The aconitase activity of the samples was then determined using the following equation:

$${\text{Aconitase activity }}\left( {U/L} \right) = \frac{{\left( {A_{565} } \right)_{30} - \left( {A_{565} } \right)_{10} }}{{T \left( {{\text{min}}} \right){\text{* slope }}\left( {{\mu M}^{ - 1} } \right)}}*n$$

(*A*_565_)_10_ is the absorption measured after 10 min, (*A*_565_)_30_ is the absorption after 30 min, *T* is the reaction time in minutes and *n* is the dilution factor. The slope value was determined from the previously generated standard curve. Finally, the values for the aconitase activity were normalized to the highest value.

## Results and discussion

### Influence of expression strain on protein levels

In theory, the expression levels of the Fe–S proteins should be similar to proteins without metal cofactors. However, the Fe–S cluster has an impact on the stability of the protein, which may lead to degradation or misfolding of the protein. Thus, the amount of soluble protein depends on the correct incorporation of the Fe–S cluster. Therefore, we compared the protein levels obtained using different plasmids and cell strains.

Gene constructs for *ispH*, *nadA*, and *acnB* were designed to result in proteins with an N-terminal affinity tag consisting of ten histidine residues (His_10_) followed by the recognition site for the TEV protease to allow for removal of the affinity tag. In addition, we have also investigated the expression of the constructs pQE30-*ispH* [25] with an affinity tag consisting of six histidines (His_6_) and no protease recognition site as well as pET46-*nadA* with a His_6_ affinity tag and an enterokinase cleavage site. The latter constructs were included in the study, as they were used for protein production in previous studies. The genes of interest in this study were engineered to contain a His_10_-tag for improved binding affinity between the poly His-tag and the immobilized nickel ions, which is proportional to the length of the His-tag resulting in a tenfold higher binding affinity achieved with His_10_-tags compared to His_6_-tags [51, 52].

In this work, we use *E. coli* as a host organism, as the advantages of this organism are well known: (i) it has unparalleled fast growth kinetics in glucose-salts media with a doubling time of about 20 min given the optimal environmental conditions [53]. (ii) High cell density cultures are easily achieved [54]. (iii) Rich complex media can be made from readily available and inexpensive components. Alternatively, media in form of powder and ready-to-use liquid is commercially available. (iv) Transformation with exogenous DNA is fast and easy [55].

We have selected BL21(DE3) cell strains as they have favorable genetic characteristics, and two variants of these cells were previously genetically engineered to optimize the maturation of Fe–S proteins in vivo. BL21 cells are deficient in the Lon protease, which degrades many foreign proteins [56]. Another gene missing from the genome of the ancestors of BL21 is the one coding for the outer membrane protease OmpT, whose function is to degrade extracellular proteins. The presence of OmpT is problematic in the expression of a recombinant protein as, OmpT may digest the protein of interest after cell lysis [57]. In addition, the *hsd*SB mutation already present in the parental strain of BL21 cells is preventing plasmid loss. When the gene of interest is placed under a T7 promoter, then T7 RNA polymerase should be provided. In the popular BL21(DE3) strain, the λDE3 prophage was inserted in the chromosome of BL21 and contains the T7 RNA polymerase gene under the *lac*UV5 promoter. The *lac* promoter is the key component of the *lac* operon, which is widely used in expression vectors [58]. Lactose or molecular mimics cause induction of the system and these inducers can be used for protein production.

BL21(DE3) have been routinely used to express Fe–S proteins, as they harbor the ISC and SUF iron–sulfur cluster assembly machineries [59, 60]. However, it was previously reported that BL21(DE3) cells have a nonfunctional Suf pathway [7]. When overexpressing genes to an unusual high level, the endogenous enzymes involved in biosynthesis are not capable of producing and transferring sufficient Fe–S clusters to the target proteins. Thus, different strategies have been developed to increase the level of the Fe–S assembly machineries and the efficiency of Fe–S cluster incorporation in recombinantly produced proteins. An additional plasmid harboring the Fe–S assembly machineries ISC [25] or SUF [26] can be co-transformed into BL21(DE3) cells, which should result in increased levels of Fe–S biogenesis and transfer. Another approach to increase the level of Fe–S biosynthesis proteins is to delete the regulator of the ISC pathway, IscR, which is responsible for the attenuation of the ISC proteins. The *E. coli* BL21(DE3) *∆iscR* cell strain shows an upregulation of *isc* operon expression, leading to higher levels of the ISC proteins and the ability to maturate abnormally high levels of Fe–S proteins. Notably, previous work suggested a more general role of IscR in the regulation of Fe‐S cluster biogenesis, therefore the deletion of *iscR* may also influence the SUF protein levels [61]. However, it was reported that *E. coli* BL21(DE3) has a nonfunctional SUF pathway, therefore the deletion of the *iscR* gene in the BL21(DE3) ∆*iscR* cell strain has no impact on the SUF pathway [7]. This study also reports an engineered “SufFeScient” derivative of BL21(DE3) with a restored SUF pathway combined with enhanced *suf* operon, referred to as Suf^++^.

Previous structural studies of IspH protein report the successful gene expression using a pQE30 vector in XL1-Blue cells [29, 62]. The genetic characteristics of XL-1 Blue cells are unfavorable for gene expression and our results are not reflecting the previous observation (Fig. S1).

**Fig. 2:**
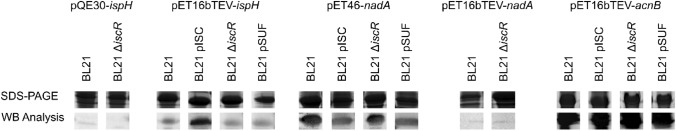
SDS-PAGE and Western blot analyses of IspH, NadA, and AcnB protein levels obtained using different expression systems. The cell lysate was analyzed by SDS-PAGE on a 15% Tris–glycine gel and proteins were visualized by Coomassie staining and the Western blot analysis using a conjugated anti-histidine antibody. A full-size image of the SDS-PAGE gels is shown in Fig. S1 and the Western blot analysis is shown in Fig. S2

We introduced each plasmid into XL1-Blue, BL21(DE3), BL21(DE3) ∆*iscR*, or BL21(DE3) Suf^++^ cells by transformation. In addition, we co-transformed all plasmids with pISC or pSUF into BL21(DE3) and analyzed the protein levels using SDS-PAGE (Figs. 2 and S1) and Western Blot analysis (Figs. 2 and S2). All plasmids, except pQE30-*ispH*, were successfully co-transformed in BL21(DE3) with either pISC or pSUF.

SDS-PAGE analysis indicates that expression using the pQE30-*ispH* plasmid results in moderate protein yields in BL21(DE3) and BL21(DE3) ∆*iscR* cells. However, the bands observed by Western Blot analysis at the appropriate position are very faint. We speculate that the His_6_-tag of the protein produced using the plasmid pQE30-*ispH* results in signals with lower intensity compared to the His_10_-tag of the protein produced using the plasmid pET16bTEV-*ispH.* Expression of *ispH* in the pET16bTEV vector shows an overexpression of the gene in all derivatives of BL21(DE3) and Western blot analysis confirms that the proteins contain a His_10_-tag. Notably, the intensity of the signal on the Western blot does not necessary correspond to the amount of protein, since there are several factors that influence visualization.

Expression of pET46-*nadA* in all BL21(DE3) cell strains resulted in good protein levels as determined by SDS-PAGE. Co-transformation of pET16bTEV-*nadA* with an additional plasmid into BL21(DE3) or transformation into BL21(DE3) Suf^++^ were not successful, thus, we were only able to investigate the expression in BL21(DE3) and BL21(DE3) ∆*iscR.* SDS-PAGE indicates a low protein yield in BL21(DE3) and a considerably high protein yield in BL21(DE3) ∆*iscR* cells. However, Western blot analysis shows very weak signals for both cell strains. SDS-PAGE and Western blot analysis of cell lysates showed high protein yields for the expression of *acnB* on the plasmid pET16bTEV in all derivatives of BL21(DE3). Taken together, we obtained the best results using the plasmids pET16bTEV-*ispH*, pET46-*nadA*, and pET16bTEV-*acnB* in all derivatives of BL21(DE3). Thus, we have used these plasmids for an in-depth investigation of the different Fe–S maturation strategies.

### Analysis of Fe–S content

We investigated the Fe–S cluster occupancy and the metal content of the produced proteins using a combination of electronic absorption spectroscopy, ECD spectroscopy, and mass spectrometry. Absorption bands in the electronic spectrum at 325 nm and in the range of 400–420 nm are a strong indicator for protein-bound Fe–S clusters. These spectral features derive from S-to-Fe charge transfer transitions with typical molar extinction coefficients of ~ 10^3^–10^4^ M^−1^ cm^−1^ [63]. CD spectroscopy measures the difference in the absorption of left and right circularly polarized light, which leads to a splitting of the features in maxima and minima. This results in an improved resolving power for overlapping absorption bands and are, therefore, particularly useful for the broad absorption features of Fe–S proteins [1]. In addition to the qualitative analysis by spectroscopic methods, we determine the metal content utilizing ICP-MS measurement. The metal content of a protein allows for quantification of the protein fraction that binds the Fe–S cofactor. The combination of these techniques provides insights into the Fe–S cluster occupancy to the protein of interest.

**Fig. 3:**
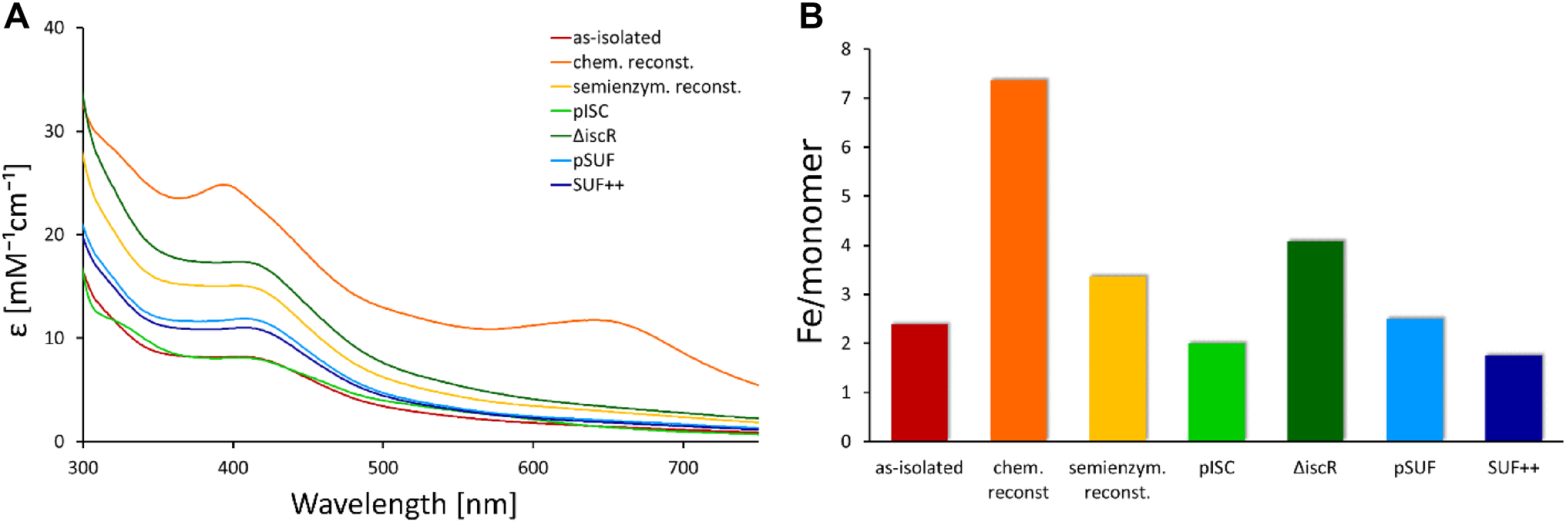
Analysis of Fe–S cluster occupancy of IspH protein obtained by different strategies. **A** EAS of IspH protein from different cell strains and after Fe–S reconstitution reactions. Corresponding ECD spectra are shown in Fig. S5. **B** Iron content per monomer of the samples determined by ICP-MS measurements. Protein as-isolated from *E. coli* BL21(DE3) is shown in red, samples obtained by subsequent chemical or semi-enzymatic reconstitution are displayed in orange and yellow, respectively. Samples co-expressed with pISC are shown in green, protein isolated from BL21(DE3) ∆*iscR* is presented in dark green. Samples co-expressed with pSUF are shown in blue, protein isolated from BL21(DE3) Suf^++^ is presented in dark blue

The first protein that we investigated in terms of Fe–S cluster maturation is *E. coli* IspH. Three protein samples were isolated from different derivatives of BL21(DE3), two samples were obtained by co-expression with either pISC or pSUF, and two samples were isolated from BL21(DE3) cells and subsequently maturated using either chemical or semi-enzymatic reconstitution. The proteins were isolated using IMAC and the purity of the samples was assessed by SDS-PAGE (Fig. S3). The electronic spectra of these protein samples are dominated by an absorption band at 415 nm (Fig. 3A), which is in agreement with previous studies [29, 64]. However, the molar extinction coefficients are different for each expression system. Protein maturated by chemical reconstitution leads to the highest molar absorption coefficient at 400 nm with a value of 25 mM^−1^ cm^−1^, which is considerably higher than the values reported in the literature [63]. Extinction coefficients of 10 mM^−1^ cm^−1^ are commonly reported for Fe–S proteins with fully occupied cofactor. In addition to the characteristic feature around 400 nm, the electronic spectrum of the chemically reconstituted protein shows a large feature at 650 nm, which indicates the presence of impurities. In line with this observation is the high iron content of seven Fe/monomer as determined by ICP-MS (Fig. 3B), which suggests the presence of unspecifically bound metals.

Expression in BL21(DE3) ∆*iscR* cells leads to an absorption maximum at 415 nm with an extinction coefficient of 17 mM^−1^ cm^−1^ and four Fe/monomer, which is in good agreement with a fully occupied [4Fe–4S] cluster in the active site of IspH. No additional band is observed at higher wavelengths, indicating no metal impurities. We have isolated and characterized the protein IspH from BL21(DE3) ∆*iscR* cells in three independent experiments to ensure the reproducibility of the high Fe–S cluster content (Fig. S4). While we observe some variation in the Fe–S cluster occupancy, the molar extinction coefficients and the iron/monomer ratio indicate a high Fe–S content. Maturation of IspH by semi-enzymatic reconstitution also results in nearly optimal cluster incorporation and the spectra shows no indication of excess metal binding. Protein obtained by co-expression with the pSUF plasmid and isolated from BL21(DE3) Suf^++^ results in a moderate Fe–S cluster occupancy.

To investigate the nature of the impurities obtained by chemical reconstitution, we measured ECD spectra [65]. The chiral nature of Fe–S clusters inside proteins makes Fe–S proteins amenable for CD spectroscopy. While all Fe–S proteins exhibit characteristics CD signals, the spectra of [4Fe–4S] cluster-containing proteins are less defined compared to [2Fe–2S] proteins and often of low intensity [66]. While the spectra of CD and electronic absorption appear to be similar, the physical origin of CD and electronic absorption are very different. The area of the absorption band is proportional to the electric dipole stretch of the transition between the ground and excited electronic states. However, to observe a CD band, the transition needs to be able to generate changes on both an electric dipole and a magnetic dipole. This requirement derives from the fact that circular polarized light excites electrons in a helical motion, requiring the electronic excitation to undergo both translational (electric dipole) and rotational (magnetic dipole) operations. Therefore, the intensities of CD signals are relatively constant across the near-UV–visible region. Low intensity or absence of a signal in the CD spectrum can also indicate a higher symmetric environment. The advantage of CD spectroscopy over EAS is that the absorption bands split up to maxima and minima, therefore, facilitating the detection of subtle changes, and bands deriving from achiral, absorbing impurities do not contribute to the spectrum. The latter includes metal ions in solution, as well as metal complexes formed with buffer substances or other reagents, such as reducing substances. The ECD spectra of the IspH protein maturated in the engineered BL21(DE3) strains ∆*iscR* and Suf^++^ or in BL21(DE3) pSUF cells (Fig. S5) show intense signals in the range of 350–450 nm, while protein obtained by chemical reconstitution displays low CD signals, indicating that maturation inside cells leads to a more efficient and specific Fe–S cluster incorporation. In addition, the broad feature at 650 nm in the electronic absorption spectrum (Fig. 3A) is not present in the ECD spectrum, suggesting that this band derives from the formation of Fe–S aggregates.

To verify that the Fe–S impurities after chemical reconstitution are unspecific Fe–S aggregates and not bound to the protein, we performed size exclusion chromatography (SEC) on this sample. The electronic spectrum (Fig. S6) of the protein after the SEC reveals that the absorption feature at 650 nm is completely removed by this additional purification step. Thus, we conclude that chemical reconstitution of Fe–S clusters leads to the formation of Fe–S aggregates with an intense absorption at 650 nm, which was not observed in any other sample.

Several expression strategies have previously been reported for IspH from *E. coli* as well as *Aquifex aeolicus*. For this purpose, expression was performed in *E. coli* M15, *E. coli* TOP10, and *E. coli* BL21(DE3) cells [64, 67, 68]. These studies reported by three different groups all reported very low Fe–S content, with very low A_410_/A_280_ ratios, which they increased by chemical reconstitution. A different study reported the co-expression of *E. coli* IspH protein with the pISC plasmid in *E. coli* XL1-Blue cells [29]. Notably, these experiments were carried out using a pISC plasmid with several point mutation that were only discovered afterwards [69]. We have tried to reproduce these results using a pISC plasmid with the correct sequences, however, no expression was detectable by Western blot analysis (Fig. S2).

**Fig. 4:**
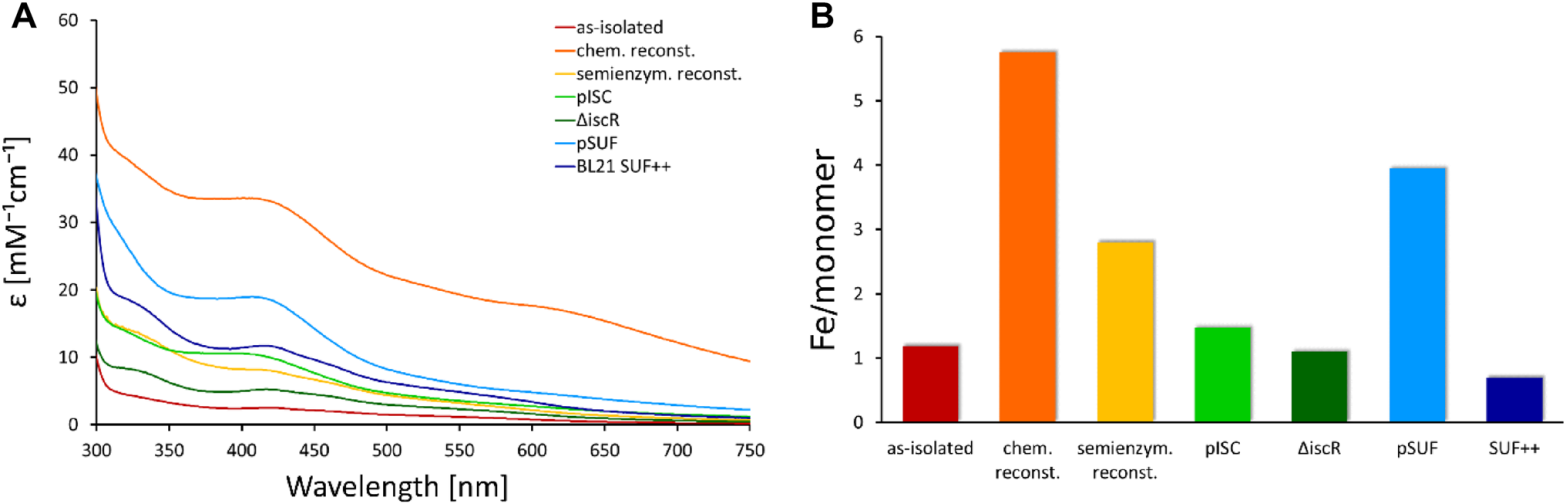
Characterization of Fe–S cluster occupancy of NadA protein obtained by different strategies. **A** EAS of protein from different cell strains and after Fe–S reconstitution reactions. Corresponding ECD spectra are shown in Fig. S7. **B** Iron content per monomer of the samples determined by ICP-MS measurements. Protein as-isolated from *E. coli* BL21(DE3) is shown in red, samples obtained by subsequent chemical or semi-enzymatic reconstitution are displayed in orange and yellow, respectively. Samples co-expressed with pISC are shown in green, protein isolated from BL21(DE3) ∆*iscR* is presented in dark green. Samples co-expressed with pSUF are shown in blue, protein isolated from BL21(DE3) Suf^++^ is presented in dark blue

The second protein that we investigated is the [4Fe–4S] cluster-containing protein quinolinate synthetase NadA. Our spectroscopic analysis of the NadA protein samples reveals that the trends observed for IspH are not reproduced in these experiments. As for IspH, protein maturated by chemical reconstitution leads to a high absorption band at 415 nm with a molar extinction coefficient of 33 mM^−1^ cm^−1^, a broad feature beyond 600 nm (Fig. 4A) and a Fe/monomer ratio of six (Fig. 4B), suggesting the formation of Fe–S aggregates. In contrast, co-expression of *nadA* with the pSUF plasmid results in an electronic spectrum with a single feature at 415 nm with a molar extinction coefficient of 19 mM^−1^ cm^−1^, indicating a high Fe–S cluster occupancy without any impurities. The Fe/monomer ratio of four as determined by mass spectrometry leads to the conclusion that this strategy produces a fully maturated Fe–S protein sample. The Fe/monomer ratio of three for the protein isolated from BL21(DE3) cells and maturated by semi-enzymatic reconstitution indicates a high Fe–S cluster content that is not in agreement with the moderate molar extinction coefficient at 410 nm. This indicates the presence of Fe impurities in this sample. Samples isolated from BL21(DE3), BL21(DE3) Suf^++^, BL21(DE3) ∆*iscR*, or BL21(DE3) pISC cells contain one Fe/monomer suggesting an incomplete cluster incorporation. The shifts in the region of 400–450 nm for some of the incompletely maturated samples may derive from partially occupied clusters. In summary, our results are in good agreement with the presence of an [4Fe–4S] cluster in NadA protein and show that specific and efficient maturation of NadA was achieved using the co-expression strategy with the pSUF plasmid [68]. The ECD spectra (Fig. S7) of all samples except the sample isolated from BL21(DE3) show CD bands in the range of 410–420 nm; however, their intensities are much lower compared to IspH, which could indicate a more symmetric environment of the cluster.

Chemical reconstitution of NadA protein resulted in excess metal binding (5.8 Fe atoms/monomer), which is in line with a previous study that obtained NadA by co-expression with the plasmid pDB1282, which contains the *isc* operon from *Azotobacter vinelandii* in *E. coli* BL21(DE3) followed by chemical reconstitution, resulting in 7.6 ± 0.9 Fe atoms/monomer [33]. However, other studies have isolated NadA protein containing 3.1–3.6 Fe atoms/monomer from BL21(DE3) [70], OverExpress C43(DE3) [71], or OverExpress C41(DE3) [72] cells. The strain C41(DE3) was derived from BL21(DE3). This strain has at least one mutation, which prevents cell death associated with expression of many recombinant toxic proteins. The strain C43(DE3) was derived from C41(DE3) by selecting for resistance to a different toxic protein and can express a different set of toxic proteins to C41(DE3). Cell strains that are effective in expressing toxic proteins may be more suitable for expression of Fe–S proteins. These studies by the same group also reported 3.1 Fe atoms/monomer for protein isolated from BL21(DE3) cells [70], which is significantly higher than the 1.2 Fe atoms/monomer that we have observed. This discrepancy most likely derives from the different conditions for gene expression. While the previous study used lysogeny broth (LB) media, induced gene expression at an OD_600_ of 0.5, and harvested the cells after 3 h at 37 °C, our protocol used a richer media (2YT or TB), in which the cell cultures reach the mid-log phase at an OD_600_ of 2, and harvested the cells after 20 h at 25 °C. The higher OD_600_ leads to a higher yield in terms of cell mass and the incubation for 20 h at 25 °C resulted in higher amounts of protein in our study. Using LB media or reducing the time span for protein expression may result in lower protein yields, enabling the Fe–S biogenesis machineries of the BL21(DE3) cells to maturate a larger fraction of the proteins. If the protein levels increase, the endogenous biogenesis systems are not able to keep up the maturation process.

Investigating the maturation of NadA, we find that co-expression with the SUF plasmid leads to the highest Fe–S cluster incorporation. This raises the question whether NadA interacts specifically with the SUF machinery inside the cell? To our knowledge, the interaction of NadA with the SUF proteins has not yet been investigated in detail. Previous studies of *E. coli* NadA with an *E. coli* strains lacking the *iscS* gene have shown that NAD biosynthesis is impaired, leading to the conclusion that *iscS* is essential for the maturation of NadA [73]. Apparently, *sufS* was not able to replace *iscS* in these experiments and maturate NadA protein. This hypothesis has not been further investigated on a molecular level. Controversially, another study reports a specific connection between NadA and a potential new system for Fe–S assembly in *E.coli* referred to as CSD [18, 74, 75]. The role of this system in Fe–S cluster biosynthesis has not been established yet and recent review articles on this topic only mention two Fe–S biosynthesis pathways in *E. coli* [14]. The only evidence for the interaction of NadA and a member of the SUF machinery was provided in *Arabidopsis thaliana* [76]. Herby, a characterization of two novel chloroplast SufE-like proteins from *A. thaliana* was reported and the results revealed that the mature SufE3 contains two domains, one SufE-like and one with similarity to the bacterial quinolinate synthase, NadA. They showed that SufE3 displayed both SufE activity and quinolinate synthase activity, thus, they concluded that SufE3 is the NadA enzyme of *A. thaliana*. A more recent study has investigated how compatible heterologous Fe–S pathways are with Fe–S proteins [49]. They have shown that co-expression with heterologous Fe–S pathways can reactivate heterologous Fe–S enzymes. Interestingly, they observed that only NadA orthologs originating from organisms harboring SUF Fe–S biogenesis pathways could be recovered by *E. coli* or *Bacillus subtilis* SUF expression. Our results support the possibility of a close relation between NadA and the SUF machinery. However, the observations for the maturation of recombinantly produced proteins may not necessarily reflect on the physiological processes inside the cell under normal conditions.

**Fig. 5:**
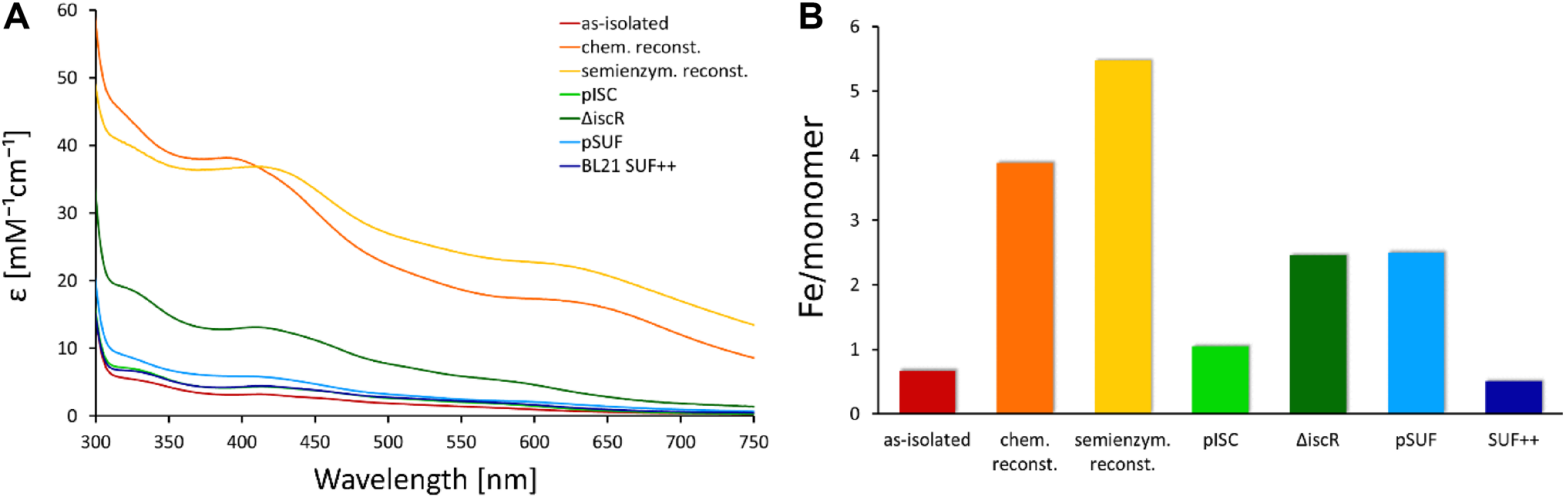
Characterization of Fe–S cluster occupancy of AcnB protein obtained by different strategies. **A** EAS of protein from different cell strains and after Fe–S reconstitution reactions. Corresponding ECD spectra are shown in Fig. S8. **B** Iron content per monomer of the samples determined by ICP-MS measurements. Protein as-isolated from *E. coli* BL21(DE3) is shown in red, samples obtained by subsequent chemical or semi-enzymatic reconstitution are displayed in orange and yellow, respectively. Samples co-expressed with pISC are shown in green, protein isolated from BL21(DE3) ∆*iscR* is presented in dark green. Samples co-expressed with pSUF are shown in blue, protein isolated from BL21(DE3) Suf^++^ is presented in dark blue

The third protein under investigation is *E. coli* AcnB, which contains an [3Fe–4S] cluster in the inactive form that can be activated by incorporation of a fourth iron resulting in the active [4Fe–4S] form [77]. Our spectroscopic analysis of AcnB produced using the previously mentioned strategies reveals significant differences in the cluster content of the different samples. Expression in BL21(DE3) cells in the absence or presence of an additional plasmid encoding the genes for the ISC or SUF machinery led to the isolation of the apo protein without significant amounts of iron as indicated by the electronic spectrum (Fig. 5A). We were able to increase the amount of Fe–S cluster in protein isolated in the apo form from *E. coli* BL21(DE3) cells using chemical or semi-enzymatic reconstitution. Both protocols led to samples with molar extinction coefficient of 40 mM^−1^ cm^−1^ for the predominant absorption band. Notably, the molar extinction coefficients are significantly higher than previously reported for *E. coli* AcnB obtained by expression in the *E. coli* strain W3110 and maturated by chemical reconstitution (8–10 mM^−1^ cm^−1^) [78]. Metal quantification using ICP-MS shows that these protein samples bind 4–6 Fe/monomer indicating the presence of some impurities in the semi-enzymatic reconstitution. For AcnB, we observe for the first time that Fe–S aggregates have been formed in the semi-enzymatic reconstitution reaction. Interestingly, we see a shift in the absorption peak between chemical and semi-enzymatic reconstitution, possibly deriving from the formation of the inactive [3Fe–4S] and the active [4Fe–4S] cluster. Spectra of AcnB obtained from *E. coli* BL21(DE3) ∆*iscR* cells exhibits an absorption band at 420 nm with a molar extinction coefficient of 14 mM^−1^ cm^−1^, which suggesting a high Fe–S clusters occupancy, however, the low Fe content as determined by ICP-MS indicates a low cluster occupancy.

Protein samples produced using BL21(DE3), BL21(DE3) pISC, BL21(DE3) pSUF, or BL21(DE3) Suf^++^ cells resulted low Fe–S cluster occupancy. For two of these samples, we observe an unexpected high Fe content, although there are no additional bands in the electronic spectrum hinting at the presence of Fe–S aggregate formation. We speculate that the discrepancy between this ICP-MS result and the EAS may be caused by inaccuracy during determining the protein concentration, which has previously been reported to be the most error-prone step [79]. In summary, our results show that reconstitution of Fe–S clusters using the chemical or semi-enzymatic approach lead to high Fe–S cluster occupancy, however, these samples contain also a larger amount of Fe–S aggregates. Assembly of Fe–S clusters in vivo is more specific and efficient than reconstitution reactions, therefore an additional purification step by SEC is not necessary.

When taking a closer look at the 400 – 450 nm range of the CD spectra (Fig. S8), we observe that the maxima of the chemically and semi-enzymatically reconstituted protein are at 410 nm, whereas the maximum of the protein isolated from BL21(DE3) ∆*iscR* cells is shifted to 450 nm. Notably, the features in the CD spectra of IspH and NadA are at 410 nm. Previously, a spectroscopic investigation of [4Fe–4S] ferredoxin from *Bacillus stearothermophilus* has shown that the peak shifts are related to a change in oxidation states [80]. The maximum at 410 nm in the ferredoxin spectrum was assigned to the oxidized form of the [4Fe–4S] cluster, whereas the maximum at 450 nm corresponds to the reduced form. We speculate that the shifts in the CD spectra could indicate different oxidation states of the [4Fe–4S] cluster in AcnB.

Taken together, our results from the systematic analysis of seven different strategies for the maturation of three different [4Fe–4S] proteins show that in vivo maturation results in more specific and effective maturation of [4Fe–4S] proteins. Each protein displays a preference for a maturation strategy that yields a high content of Fe–S cluster without any spectroscopically detectable excess iron species. However, the suitable biosynthesis method must be determined experimentally for each protein. In all three cases, chemical reconstitution leads to high Fe–S cluster occupancy in the protein, but also to the formation of high amounts of Fe–S aggregates, which require an additional SEC purification step to separate the impurities from the protein. In one case, we have also observed that maturation by reconstitution results in a different oxidation state of the [4Fe–4S] cluster compared to in vivo maturation. ECD spectroscopy has been a very useful tool to obtain more detailed information about the Fe–S cluster and provided insights on the oxidation states of the cluster.

In this study, we characterized the proteins directly after the isolation from the host organism using IMAC. The proteins produced using the pET16bTEV plasmid contain a TEV recognition site between the His_10_-tag and the protein of interest. As His-tags have a high affinity for divalent metal ions, they can interfere with reconstitution reactions. Zinc inhibits the activity of the TEV protease, and DTT and EDTA are added to the reaction to ensure high reactivity. Thus, TEV cleavage will likely also lead to the degradation of Fe–S clusters. Therefore, we have not routinely removed the His_10_-tag in this work. However, we aimed at testing whether the His_10_-tag affects the reconstitution reactions. AcnB protein isolated from BL21(DE3) by IMAC was treated with TEV protease to remove the His_10_-tag followed by reconstitution of the Fe–S cluster using either the chemical or semi-enzymatic approach. The electronic spectra (Fig. S9) indicate a dramatically decreased Fe–S cluster content in AcnB, suggesting that most of the protein is in its apo form. We speculate that exposing AcnB to a combination of DTT and EDTA led to misfolding of the protein, rendering it unable to bind an Fe–S cluster. So far, we have investigated three proteins, AcnB, IspH, and NadA, which contain only one [4Fe–4S] cluster and have previously been characterized. Next, we aimed at transferring the new insights on Fe–S cluster maturation on the radical SAM enzyme ThnB, which has not yet been maturated in vivo.

### Maturation of the radical SAM enzyme ThnB

The radical SAM enzyme ThnB from *B. thuringiensis* has been reported to host two [4Fe–4S] clusters and is required for thioether bond formation during the maturation of the sactipeptide ThnH [38]. All previous studies were performed on protein obtained by recombinant production in *E. coli* BL21(DE3) directed from the expression plasmid pET28a(+). In the present study, we directed the expression of the *thnB* gene from the expression plasmid pET16bTEV and investigated the same expression and maturation protocols as for the three well-characterized samples.

SDS-PAGE and Western blot analysis of the expression levels in *E. coli* BL21(DE3) in the presence and absence of the plasmids pISC or pSUF, in BL21(DE3) *∆iscR* (Fig. S10A and B) and BL21(DE3) Suf^++^ (Figs. S1 and S2) show that the co-expression of *thnB* with the pSUF plasmid results in the highest amount of protein. A slightly lower expression level has been detected for the co-expression with pISC and the expression in BL21(DE3) *∆iscR* or BL21(DE3) Suf^++^*.* In line with our previous observations, expression in BL21(DE3) results in the lowest protein levels. These results show that specific expression systems tailored for Fe–S proteins not only increase the fraction of Fe–S cluster-containing protein, but also lead to higher protein levels. The purity of the ThnB samples is lower than for the other proteins of interest (Fig. S9C), however, an additional purification step was not introduced to be able to compare these data with the previous ones.

**Fig. 6:**
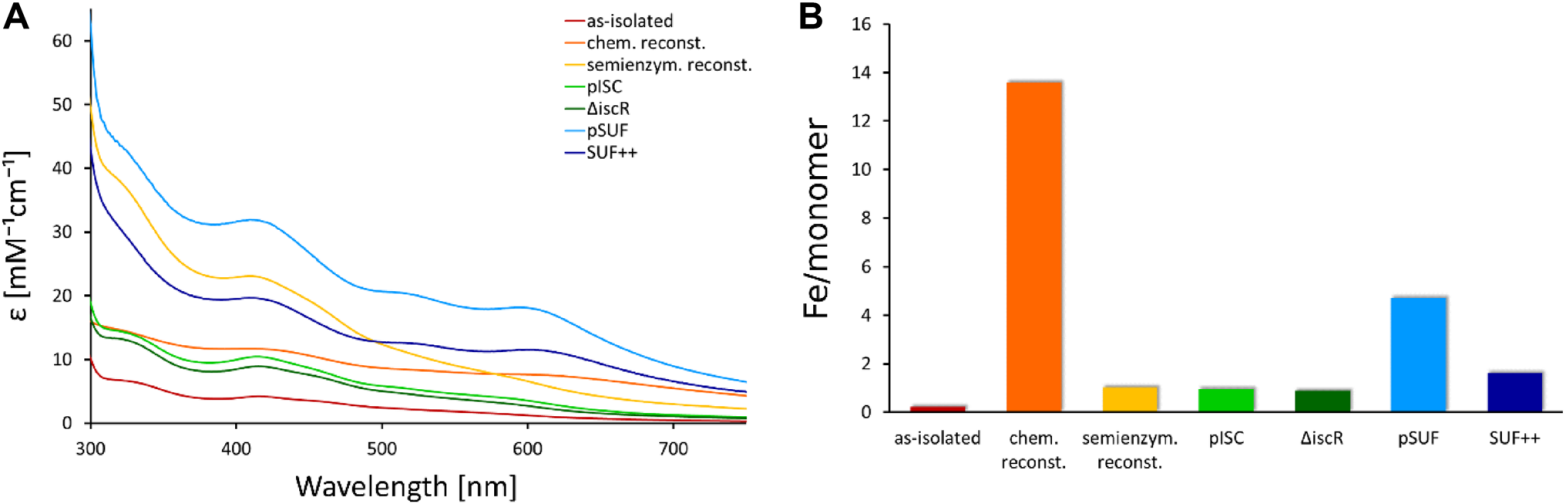
Analysis of Fe–S cluster occupancy of ThnB protein obtained by different strategies. **A** EAS of ThnB protein from different cell strains and after Fe–S reconstitution reactions. Corresponding ECD spectra are shown in Fig. S11. **B** Iron content per monomer of the samples determined by ICP-MS measurements. Protein as-isolated from *E. coli* BL21(DE3) is shown in red, samples obtained by subsequent chemical or semi-enzymatic reconstitution are displayed in orange and yellow, respectively. Samples co-expressed with pISC are shown in green, protein isolated from BL21(DE3) ∆*iscR* is presented in dark green. Samples co-expressed with pSUF are shown in blue, protein isolated from BL21(DE3) Suf^++^ is presented in dark blue

The electronic spectra (Fig. 6A) show that co-expression with pSUF leads to the highest Fe–S cluster occupancy. The Fe/monomer ratio of ThnB obtained by co-expression with pSUF is 5.3 iron per monomer (Fig. 6B), which is lower than expected for a protein binding two [4Fe–4S] clusters. The iron per monomer ratio is only as accurate as the metal and protein concentrations. The metal content was measured using ICP-MS, which is a very precise technique, while the determination of the protein concentration is prone to larger errors. We have determined the protein concentration using the Bradford method [50]. Considering that the purity is estimated between 60 and 80 % based on SDS-PAGE (Fig. S9C), the actual ThnB concentration is lower than calculated, resulting in an underestimation of the iron per monomer value. Also taking the molar extinction coefficient at 413 nm or 32 mM^−1^ cm^−1^, we can assume that the protein obtained from co-expression with pSUF is fully maturated. Another possible explanation would be that the protein contains one [2Fe–2S] cluster in addition to a [4Fe–4S] cluster. The electronic spectrum of the sample obtained from co-expression contains additional absorption bands at 515 nm and 615 nm. A previous study of the antiviral radical SAM enzyme viperin has assigned the feature at 615 nm to a [2Fe–2S]^2+^ cluster [81]. Similar bands have been observed in the electronic spectrum of the active spore photoproduct lyase, a member of the radical SAM superfamily from *Bacillus* [82]. However, these features have not been analyzed in detail. The iron content (Fig. 6B) would be consistent with one [4Fe–4S] and one [2Fe–2S] cluster in ThnB. The ECD spectrum of ThnB protein obtained by co-expression of the *thnB* gene with the pSUF plasmid shows a maximum at 450 nm, which indicates that the two [4Fe–4S] clusters are isolated in the reduced form (Fig. S11). However, since a feature in the CD spectrum at 450 nm is also characteristic for an oxidized [2Fe–2S] cluster, we cannot exclude the possibility of a [2Fe–2S] cluster bound to ThnB.

To remove protein impurities and to investigate whether the impurities resulting in absorption bands at 515 nm and 615 nm can be separated by further purification steps, we have performed SEC on a protein sample isolated from BL21(DE3) pSUF (Fig. S12). The spectrum reveals a low molar extinction coefficient of 0.03 mM^−1^ cm^−1^ after the SEC, which is significantly lower than before this purification step (30 mM^−1^ cm^−1^), indicating that the majority of the cluster has degraded. Interestingly, we still observe very weak bands at 515 nm and 615 nm, which suggest that the species giving rise to these features cannot be separated by SEC and might be a feature of the protein-bound cofactors.

The electronic spectra of protein produced in BL21(DE3) cells followed by maturation using semi-enzymatic reconstitution or isolated from BL21(DE3) Suf^++^ cells suggest a good cluster occupancy; however, ICP-MS measurements (Fig. 6B) suggest that the protein binds less than one Fe/monomer. Expression in BL21(DE3) *∆iscR* cells, co-expression with pISC, and maturation by chemical reconstitution leads to a low Fe–S cluster content, while expression in BL21(DE3) cells results in apo ThnB. The spectroscopic observations are consistent with the iron content determined by ICP-MS, except the surprisingly high iron content in the sample obtained by chemical reconstitution. This ThnB sample binds almost 14 iron per monomer, which is much higher than expected for a protein binding two [4Fe–4S] clusters and compared to the relatively low absorption band at 413 nm in the electronic spectrum with a molar extinction coefficient at 413 nm of 12 mM^−1^ cm^−1^. The molar extinction coefficient previously reported for chemically reconstituted ThnB was also considerably higher (30 mM^−1^ cm^−1^) [38]. Unfortunately, we were not able to reproduce the high Fe–S content in ThnB and speculate that the high iron content consists of excess reagents that were not removed by desalting or Fe–S aggregates.

*E. coli* harbors two machineries for Fe–S cluster biogenesis and we are just beginning to understand how these machineries interact with their target proteins. One of the remaining questions is, whether each machinery is capable to maturate the same set of proteins or whether the cell prioritizes under oxidative stress conditions. Furthermore, it remains unclear, whether one protein has a preference for a maturation system inside the cell and whether this system is able to maturate recombinantly produced proteins more efficiently than the other system. Our results do not support a clear preference for a Fe–S biogenesis system for the maturation of recombinantly produced proteins, but it shows that there is one preferred strategy for the maturation of a specific protein. In general, chemical reconstitution results in excess metal binding and may also result in a non-native cluster form, while in vivo maturation produces more homogenous protein samples.

### Enzyme activity of AcnB

**Fig. 7:**
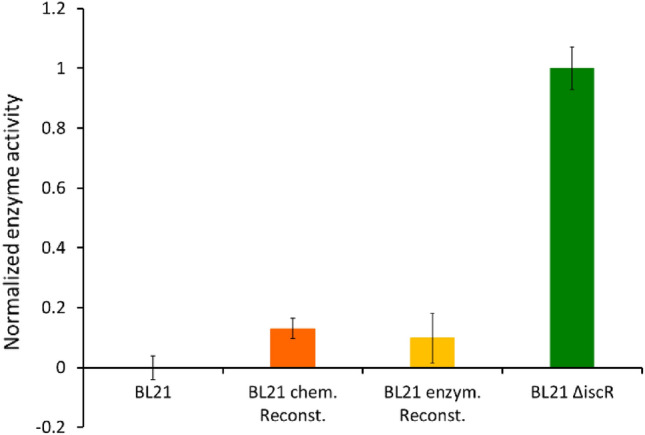
Measurements of the enzymatic activity of AcnB protein produced by different strategies. For clarity, the data have been normalized to the highest value, which was achieved with samples isolated from BL21(DE3) ∆*iscR* (dark green). Activity of the samples as-isolated from *E. coli* BL21(DE3) were below the detection limit. Results for the samples obtained by chemical or semi-enzymatic reconstitution are displayed in orange and yellow, respectively

The intact Fe–S cluster is essential for the enzymatic activity of the proteins of interest. Therefore, we have thoroughly investigated the maturation of the Fe–S proteins. We further want to evaluate if the incorporated cluster also results in a functionally active protein. We have used AcnB isolated from BL21(DE3) and BL21(DE3) Δ*iscR* as well as protein samples obtained by chemical and enzymatic reconstitution to measure the enzymatic activity (Fig. 7). Our results show the highest enzymatic activity for the protein isolated from BL21(DE3) Δ*iscR* cells. Enzymatic activity of protein isolated from BL21(DE3) cells was below the detection limit. AcnB that was maturated in vitro by chemical or semi-enzymatical reconstitution showed less than 20 % of the activity compared to protein from BL21(DE3) Δ*iscR* cells. This result is surprising, as the metal content of the samples maturated in vitro was the highest. Our findings are supported by a recent study on enzyme activity of iron–sulfur enzymes in non-native prokaryotic hosts [49]. Their findings show that co-expression of a heterologous Fe–S biogenesis pathway increases the chances of an orthologous protein to be functional in a foreign host. The activity measurements clearly show that Fe–S enzymes maturated in vivo are more active than proteins maturated by reconstitution.

## Conclusion

In this study, we investigate different strategies for the maturation of recombinantly produced Fe–S proteins. We compare seven different strategies for protein biosynthesis, including the common expression strain BL21(DE3), four specific cell strains tailored for the maturation of high levels of Fe–S proteins, and two in vitro reconstitution methods. The biosynthesis of Fe–S proteins in nature has been studied extensively; however, the production of large quantities of Fe–S proteins containing the correct physiological Fe–S cluster remains a major challenge. We have systematically evaluated the most common maturation protocols for three well-characterized [4Fe–4S] proteins. The pitfalls of chemical reconstitution pointed out here, such as the formation of Fe–S aggregates, adventitiously bound iron, and different oxidation states of the cluster, have previously led to conflicting results in different studies. These strategies are not limited to proteins containing one [4Fe–4S] cluster. We have previously shown that the same strategies can be applied for the maturation of a [2Fe–2S] protein [83]. In addition, we have demonstrated that this approach can be also used for radical SAM enzymes. The different strategies investigated in this study can be applied to various Fe–S proteins and contribute to improving the maturation of Fe–S proteins. Maturation of the proteins in vivo also leads to more homogeneous samples, which are more amenable for structural and spectroscopic experiments. Obtaining samples with high cofactor content that is in the physiologically relevant form will lead to a better understanding of the structure and function of Fe–S proteins.

## Supplementary Information

Below is the link to the electronic supplementary material.

Supplementary file1 (PDF 1021 KB)

## Data Availability

The datasets generated during the current study are available from the corresponding author on reasonable request.
