# Supplementary material for: Maturation strategy influences expression levels and cofactor occupancy in Fe–S proteins
Source: J Biol Inorg Chem. 2022 Dec 17;28(2):187–204. doi: 10.1007/s00775-022-01972-1 (PMC9981529; doi:10.1007/s00775-022-01972-1)
Supplement: Supplementary file 1 — Supplementary file1 (PDF 1021 KB) [file 775_2022_1972_MOESM1_ESM.pdf]

Supplementary Information

for

Maturation strategy influences expression levels and cofactor occupancy in Fe-S proteins

Melissa Jansing[1,†], Steffen Mielenbrink[1,†], Hannah Rosenbach[1,†], Sabine Metzger[2], and Ingrid Span[1,3,*]

[1] Institut für Physikalische Biologie, Heinrich-Heine-Universität Düsseldorf, Universitätsstr. 1, 40225 Düsseldorf, Germany

[2] MS-Platform Biocenter, Cluster of Excellence on Plant Science (CEPLAS), University of Cologne, Zülpicher Strasse 47b, 50674 Cologne, Germany

[3] Bioanorganische Chemie, Department Chemie und Pharmazie, Friedrich-Alexander-Universität Erlangen-Nürnberg, Egerlandstr. 1, 91058 Erlangen, Germany

[†] Authors contributed equally.

[*] Corresponding author: Ingrid Span, ingrid.span@fau.de

ORCID: Melissa Jansing (0000-0002-5273-9910), Hannah Rosenbach (0000-0001-8488-3784), Steffen Mielenbrink (0000-0002-3766-3276), Sabine Metzger (0000-0002-9722-8495), Ingrid Span (0000-0002-2892-4825)

Supplementary Table

Table S1 Plasmids used in this study.

Plasmid	Reference
pACYC*iscS-fdx* (pISC)	Gräwert et al., 2004 [1]
pACYC-Duet-1-*suf* (pSUF)	Hänzelmann and Schindelin, 2004 [2]
pET16bTEV-*acnB*	This work
pET16bTEV-*ispH*	This work
pQE30-*ispH*	Gräwert et al., 2010 [3]
pET46-*nadA*	Gift from Prof. Dr. Eric Oldfield (UIUC)
pET16bTEV-*nadA*	This work
pET16bTEV-*thnB*	This work

Table S2 Cell strains used in this study.

Strain	Genotype	Reference
XL1 Blue	*recA1*, *endA1*, *gyrA96*, *thi-1*, *hsdR17*, *supE44*, *relA1*, *lac*, [*F´*, *proAB*, $lacI^{q}Z\Delta M15$, *Tn10* (tet^{r})]	Bullock et al.
BL21 (DE3)	F^{-}, *ompT*, $hsdS_{B}$, (r_{B}^{-}, m_{B}^{-}), *gal*, *dcm (DE3)*	Studier et al.
BL21 (DE3) Δ*iscR*	F^{-}, *ompT*, $hsdS_{B}$, (r_{B}^{-}, m_{B}^{-}), *gal*, *dcm iscR::kan (DE3)*	Schwartz et al., 2001 [4]
BL21 (DE3) Suf^{++}	BL21(DE3) but *zdh-3632::cat*, $^{-26}$ATA^{-24} bp relative to *sufA* TSS changed to $^{-26}$TAT^{-24}	Corless et al., 2019 [5]

Supplementary Figures

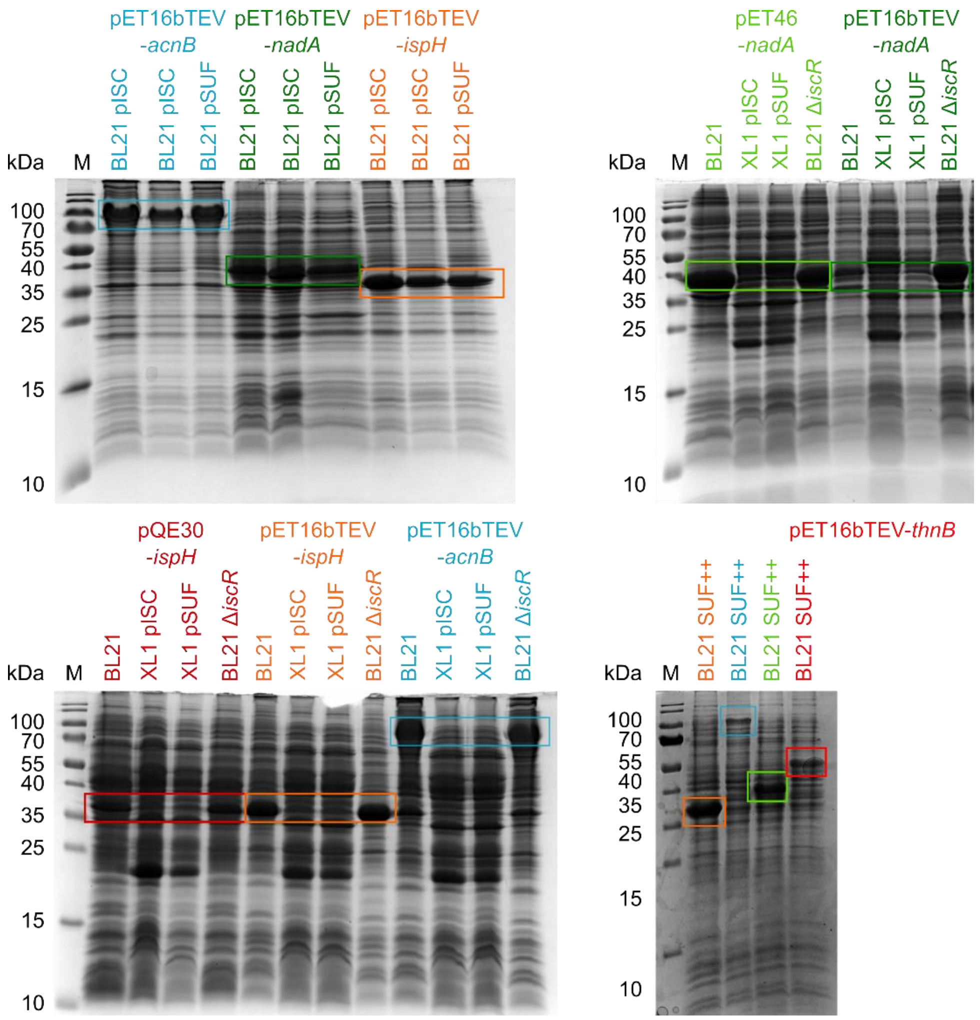

Fig. S1 Analysis of protein levels by SDS-PAGE. Protein samples were obtained using different vectors and cell strains. Samples were then normalized to an optical density of 1 at 600 nm and the same volume was applied to each lane to ensure a comparable analysis

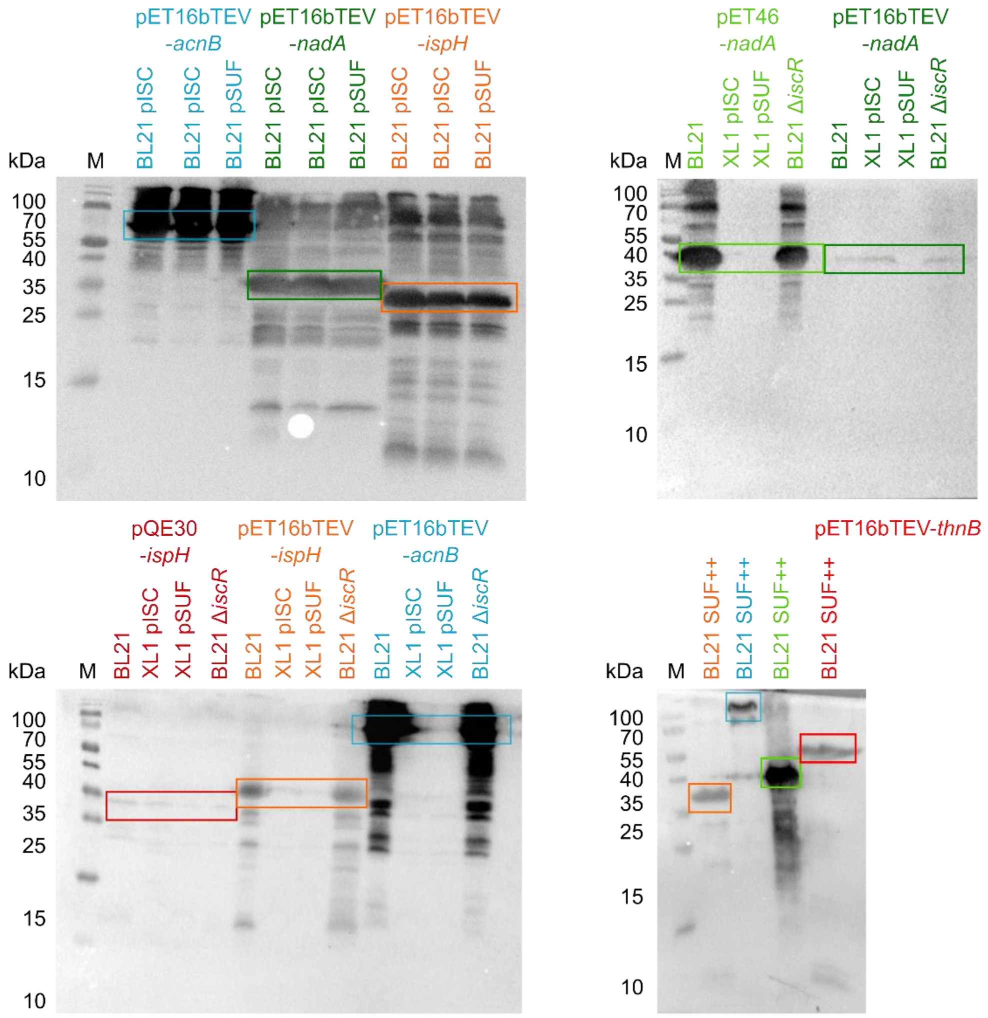

Fig. S2 Western Blot Analysis of the samples analysed by SDS-PAGE in Fig. S1. *pH*, *nadA*, and *acnB* in different vectors using SDS-PAGE. Samples were normalized to an optical density of 1 at 600 nm and the same volume was applied to each lane to ensure a comparable analysis

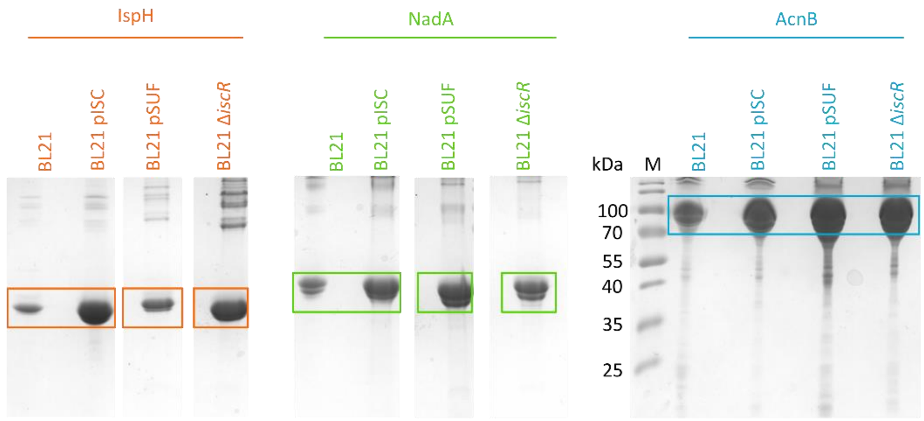

Fig. S3 SDS-PAGE analysis of purified protein samples after the first chromatography step. The protein was isolated by immobilized metal affinity chromatography using a nickel-charged affinity resin. The first step led to high purity; thus, further purification was not performed

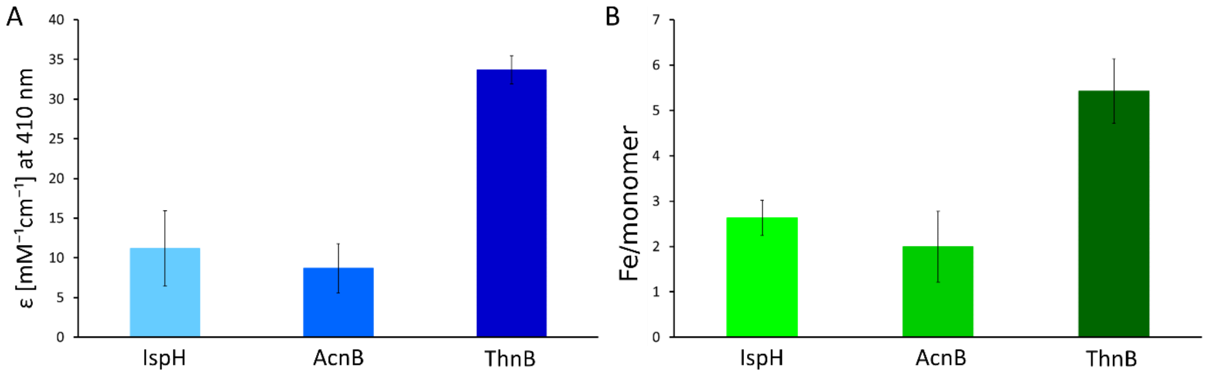

Fig S4 Average Fe-S cluster content in IspH from BL21(DE3) Δ*iscR* cells, AcnB from BL21(DE3) Δ*iscR* cells, and ThnB from BL21(DE3) pSUF cells. (A) Molar extinction coefficient measured by electronic absorption spectroscopy at 410 nm. (B) Iron/monomer ratio obtained by ICP-MS measurements. Proteins from three independent experiments were used for IspH, four independent experiments for AcnB, and two independent experiments for ThnB.

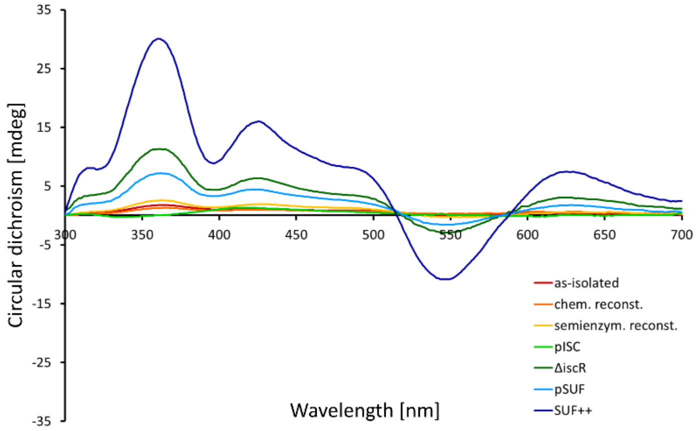

Fig. S5 Electronic circular dichroism (ECD) spectra of IspH protein. Color code: protein as-isolated in red, protein isolated from BL21(DE3) and maturated by chemical reconstitution in orange or maturated by semi-enzymatic reconstitution in yellow, protein obtained by co-expression of the *ispH* gene with the *isc* operon in green, protein obtained by expression in BL21(DE3)Δ*iscR* cells [6] in dark green, protein obtained by co-expression of the *ispH* gene with the *suf* operon in blue, and protein obtained by expression in BL21(DE3)Suf^{++} cells [5] in dark blue

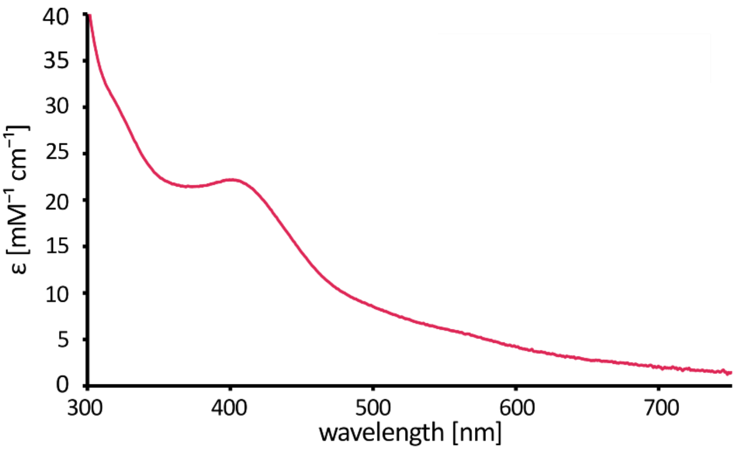

Fig. S6 Electronic spectrum of chemically reconstituted IspH after size exclusion chromatography

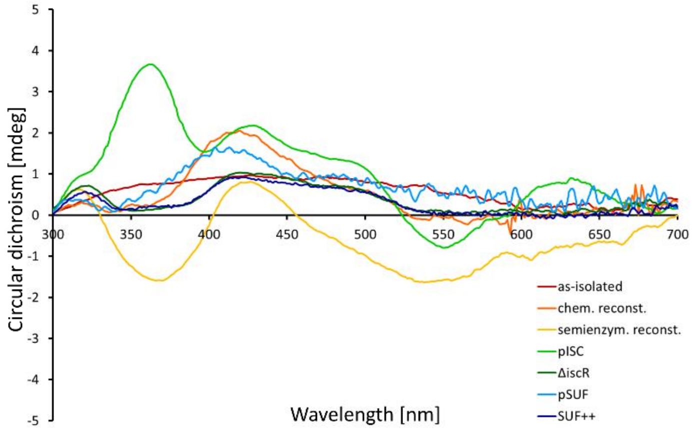

Fig. S7 ECD spectra of NadA protein. Color code: protein as-isolated in red, protein isolated from BL21(DE3) and maturated by chemical reconstitution in orange or maturated by semi-enzymatic reconstitution in yellow, protein obtained by co-expression of the *nadA* gene with the *isc* operon in green, protein obtained by expression in BL21(DE3)Δ*iscR* cells [6] in dark green, protein obtained by co-expression of the *nadA* gene with the *suf* operon in blue, and protein obtained by expression in BL21(DE3)Suf^{++} cells [5] in dark blue

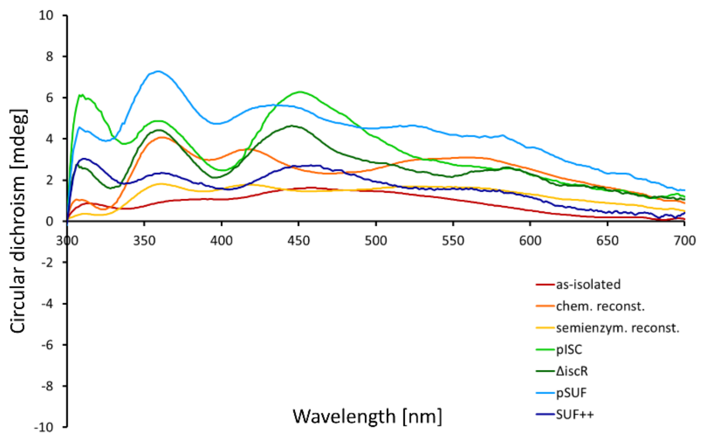

Fig. S8 ECD spectra of AcnB protein. Color code: protein as-isolated in red, protein isolated from BL21(DE3) and maturated by chemical reconstitution in orange or maturated by semi-enzymatic reconstitution in yellow, protein obtained by co-expression of the *acnB* gene with the *isc* operon in green, protein obtained by expression in BL21(DE3)Δ*iscR* cells [6] in dark green, protein obtained by co-expression of the *acnB* gene with the *suf* operon in blue, and protein obtained by expression in BL21(DE3)Suf^{++} cells [5] in dark blue

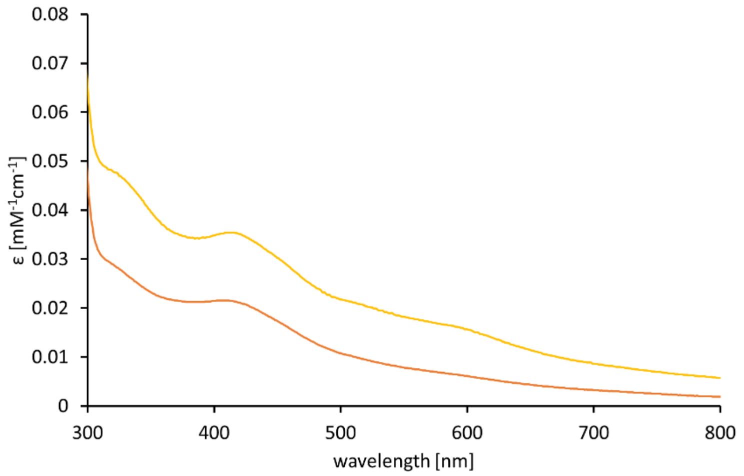

Fig. S9 Electronic spectra of AcnB protein isolated from BL21(DE3), subsequently treated with TEV protease to remove the His$_{10}$-tag, followed by chemical (orange) or semi-enzymatical (yellow) reconstitution. The molar extinction coefficient is by orders of magnitude lower compared to the protein that was reconstituted immediately after isolation, indicating that most of the protein is in the apo form.

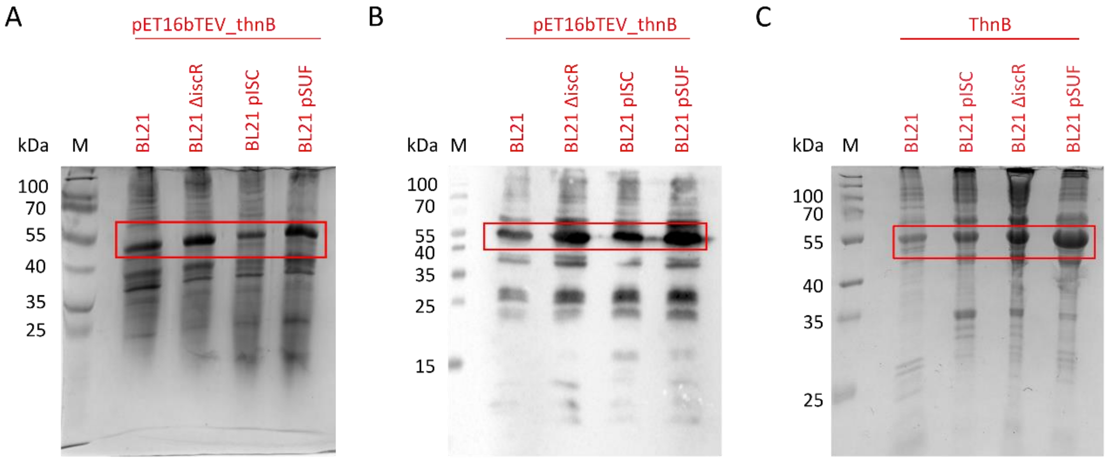

Fig. S10 SDS-PAGE and Western Blot analyses of *thnB* expression and ThnB protein after isolation by affinity chromatography. (A) SDS-PAGE analysis of the *thnB* gene expression in BL21(DE3), BL21(DE3)Δ*iscR* cells [6], BL21(DE3) pISC, and BL21(DE3) pSUF cells. Samples were normalized to an optical density of 1 at 600 nm and the same volume was applied to each lane to ensure a comparable analysis. (B) Western Blot analysis using a conjugated anti-His antibody was used to identify the target protein. (C) The protein was isolated using a nickel-loaded affinity resin and the protein samples were subsequently analyzed using SDS-PAGE

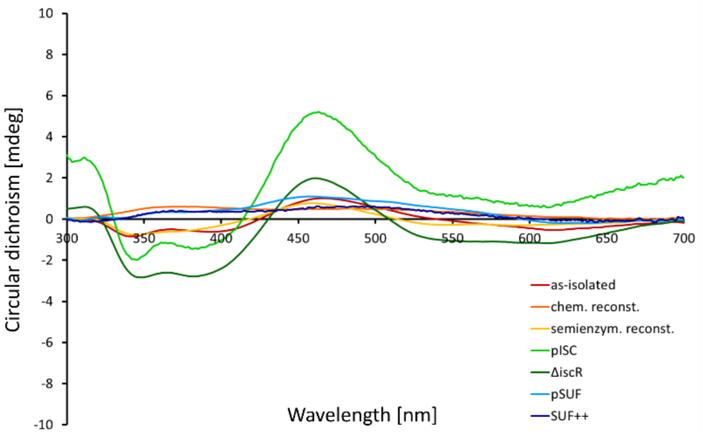

Fig. S11 ECD spectra of ThnB protein. Color code: protein as-isolated in red, protein isolated from BL21(DE3) and maturated by chemical reconstitution in orange or maturated by semi-enzymatic reconstitution in yellow, protein obtained by co-expression of the *thnB* gene with the *isc* operon in green, protein obtained by expression in BL21(DE3)Δ*iscR* cells [6] in dark green, protein obtained by co-expression of the *thnB* gene with the *suf* operon in blue, and protein obtained by expression in BL21(DE3)Suf^{++} cells [5] in dark blue

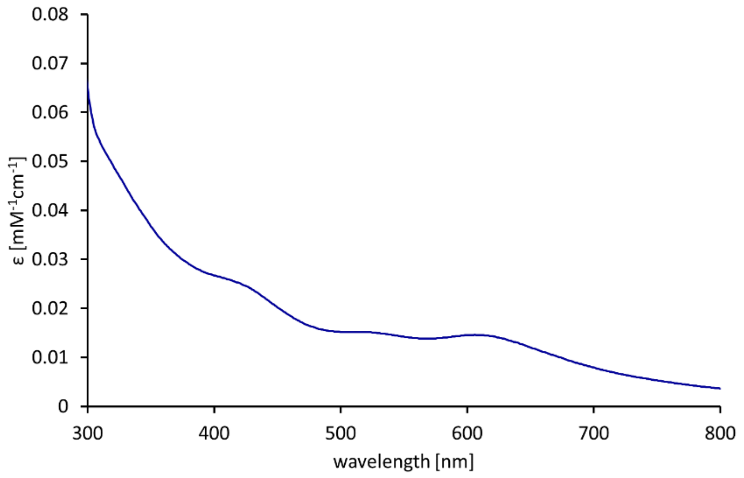

Fig. S12 Electronic spectrum of ThnB produced in BL21(DE3) pSUF, isolated by affinity chromatography and subsequently purified using size exclusion chromatography (SEC). The molar extinction coefficient when isolated from this cell strain was above 30 $mM^{-1}cm^{-1}$ and is after the SEC below 0.03, indicating that the cluster has degraded during this additional purification step

Supplementary References

1. Gräwert T, Kaiser J, Zepeck F, et al (2004) IspH protein of Escherichia coli: Studies on iron-sulfur cluster implementation and catalysis. J Am Chem Soc 126:12847–12855. https://doi.org/10.1021/ja0471727
2. Hänzelmann P, Hernández HL, Menzel C, et al (2004) Characterization of MOCS1A, an oxygen-sensitive iron-sulfur protein involved in human molybdenum cofactor biosynthesis. J Biol Chem 279:34721–34732. https://doi.org/10.1074/jbc.M313398200
3. Gräwert T, Span I, Eisenreich W, et al (2010) Probing the reaction mechanism of IspH protein by x-ray structure analysis. Proc Natl Acad Sci U S A 107:1077–1081. https://doi.org/10.1073/pnas.0913045107
4. Jaroschinsky M, Pinske C, Gary Sawers R (2017) Differential effects of isc operon mutations on the biosynthesis and activity of key anaerobic metalloenzymes in Escherichia coli. Microbiology 163:878–890. https://doi.org/10.1099/mic.0.000481
5. Corless EI, Mettert EL, Kiley PJ, Antony E (2019) Elevated Expression of a Functional Suf Pathway in Escherichia coli BL21(DE3) Enhances Recombinant Production of an Iron-Sulfur Cluster-Containing Protein. J Bacteriol 202:. https://doi.org/10.1128/JB.00496-19
6. Schwartz CJ, Giel JL, Patschkowski T, et al (2001) IscR, an Fe-S cluster-containing transcription factor, represses expression of Escherichia coli genes encoding Fe-S cluster assembly proteins. Proc Natl Acad Sci 98:14895–14900. https://doi.org/10.1073/pnas.251550898
